# Synthesis and Characterization of Covalent Triazine Frameworks Based on 4,4′-(Phenazine-5,10-diyl)dibenzonitrile and Its Application in CO_2_/CH_4_ Separation

**DOI:** 10.3390/molecules30153110

**Published:** 2025-07-24

**Authors:** Hanibal Othman, Robert Oestreich, Vivian Küll, Marcus N. A. Fetzer, Christoph Janiak

**Affiliations:** Institut für Anorganische Chemie und Strukturchemie, Heinrich-Heine-Universität Düsseldorf, 40204 Düsseldorf, Germany; hanibal.othman@hhu.de (H.O.); robert.oestreich@hhu.de (R.O.); vivian.kuell@hhu.de (V.K.); marcus.fetzer@hhu.de (M.N.A.F.)

**Keywords:** covalent triazine frameworks, BET surface area, adsorption, carbon dioxide separation from methane, heat of adsorption

## Abstract

Covalent triazine frameworks (CTFs) have gained recognition as stable porous organic polymers, for example, for CO_2_ separation. From the monomer 4,4′-(phenazine-5,10-diyl)dibenzonitrile (pBN), new pBN-CTFs were synthesized using the ionothermal method with a variation in temperature (400 and 550 °C) and the ZnCl_2_-to-monomer ratio (10 and 20). N_2_ adsorption yielded BET surface areas up to 1460 m^2^g ^−1^. The pBN-CTFs are promising CO_2_ adsorbents and are comparable to other benchmark CTFs such as CTF-1 with a CO_2_ uptake of pBN-CTF-10-550 at 293 K of up to 54 cm^3^ g^−1^ or 96 mg g^−1^, with a CO_2_/CH_4_ IAST selectivity of 22 for a 50% mixture of CO_2_/CH_4_. pBN-CTF-10-400 has a very high heat of adsorption of 79 kJ mol^−1^ for CO_2_ near zero coverage in comparison to other CTFs, and it also stays well above the liquefaction heat of CO_2_ due to its high microporosity of 50% of the total pore volume.

## 1. Introduction

**Figure 1:**
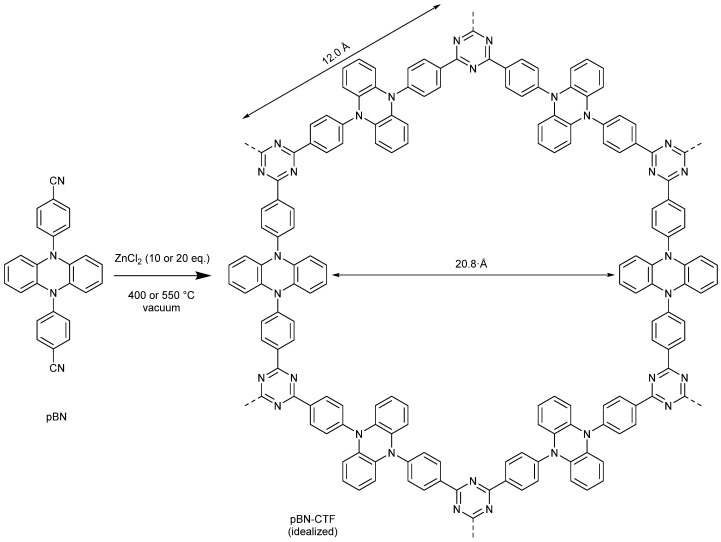
Synthesis of pBN-CTF from the monomer 4,4′-(phenazine-5,10-diyl)dibenzonitrile (pBN) with the CTF shown as an idealized hexagonal ring structure. The edge length and width of the ideal hexagon were determined graphically on the basis of the length of the C = C double with 1.34 Å.

Porous materials contain interconnected pores which can have different length scales from micro- (<2 nm) and meso- (2–50) nm to macropores (>50 nm) [1,2]. Covalent triazine frameworks (CTFs) are micro-mesoporous organic polymers that are constructed from 1,3,5-triazine rings joined with linkers to ideally give two-dimensional networks with hexagonal openings (Figure 1) [3,4,5].

The nitrogen content and the porosity of the CTFs together with their thermal stability make them interesting materials for gas adsorption, storage and separation, pollutant removal, catalysis, and sensing [6,7,8,9,10,11,12,13], including CO_2_/N_2_ and CO_2_/CH_4_ separation, both in the neat form [14,15,16,17] and as a filler for organic polymers in mixed-matrix membranes [18,19,20]. Since the inception of covalent triazine frameworks in 2008 [21], these materials have been investigated for their CO_2_ adsorption [22,23,24,25,26,27,28,29,30,31].

One of the standard synthesis methods of CTFs is the ionothermal method, in which the nitrile monomer is heated with excess zinc chloride under vacuum or an inert atmosphere to a chosen temperature in the range between 400 and 900 °C [32]. In this reaction, the molten zinc chloride salt acts as a solvent, as a Lewis acid, and as a porogen [33].

The surface area is not necessarily the determining and main property of CTFs for CO_2_ adsorption. A study showed an inverse relationship between BET surface area and the uptake of CO_2_ [16]. This correlation can be understood by the increase in the surface area with synthesis temperature and the concomitant decrease in the nitrogen content. When the CTFs are synthesized under relatively low temperatures (e.g., at 350 °C), the nitrogen loss is minimized but so is the surface area. Under higher synthesis temperatures (over 500 °C), significant nitrogen loss occurs, resulting in materials that transition towards high-surface-area carbon structures with some residual nitrogen content. Often, 400 °C is chosen as a compromise between good surface area and not too high nitrogen loss. For a high CO_2_ uptake, a high nitrogen content is aimed for. The structural features and performance of CTFs are highly sensitive to the monomer structure and the salt-to-monomer ratio employed during synthesis. For instance, adjusting the ZnCl_2_ ratio can influence the degree of polymerization, porosity, and degree of graphitization, all of which affect gas sorption behavior [21].

Several studies have demonstrated the superior CO_2_ adsorption capacity and selectivity of functionalized CTFs. Gu et al. reported CTFs with notable CO_2_ uptake and selectivity, emphasizing the role that microporosity plays in enhancing gas affinity. Similarly, Buyukcakir et al. introduced charged CTFs, showing that ionic functionality can improve both CO_2_ capture and catalytic conversion, underlining the synergy between framework charge and adsorption behavior [22,23].

The introduction of electron-withdrawing or polar functional groups such as fluorine or amines has also proven effective. Perfluorinated CTFs exhibit high CO_2_ selectivity and water tolerance, while amine-modified frameworks display improved CO_2_/CH_4_ selectivity due to favorable acid–base interactions. These findings are consistent with those of Dawson et al., who emphasized the role played by targeted chemical functionalization in enhancing CO_2_ binding through dipole–quadrupole and hydrogen bonding interactions [24,26,27].

Together, these studies demonstrate that by carefully controlling synthesis parameters—including salt-to-monomer ratios—and integrating tailored functional groups, the adsorption performance of CTFs towards CO_2_ can be significantly improved.

In this work, we use the dinitrile monomer 4,4′-(phenazine-5,10-diyl) dibenzonitrile (pBN) (Figure 1) to increase the nitrogen content for CO_2_ adsorption and separation from CO_2_/CH_4_ mixtures. The CTFs from this monomer were synthesized with two ratios of ZnCl_2_ to observe the impact of the salt ratio on the surface area and two temperatures to compare the effect of the temperature on the CO_2_ and CH_4_ adsorption.

## 2. Results and Discussion

New covalent triazine frameworks with the monomer 4,4′-(phenazine-5,10-diyl)dibenzonitrile (pBN) were synthesized via the ionothermal route with molten zinc chloride at two molar ZnCl_2_/monomer ratios of 10:1 and 20:1 at three temperatures of 350 °C, 400 °C, and 550 °C, all at the reaction time of 48 h. The CTF samples are coded as pBN-CTF-xx-yyy by giving the molar ZnCl_2_/monomer ratio (xx = 10 or 20), followed by the reaction temperature (yyy = 350, 400, or 550 °C). The pBN-CTF products were obtained as black monoliths, as typically observed for CTFs [34]. We tried to remove ZnCl_2_ through washing with acidified water, as described in the literature for CTFs [34,35]. The product yields ranged from 68 to 92% (Table 1). The scanning electron microscopy images indicate the typical shard-like morphology of CTFs (Figure S1a–d, Supplementary Materials).

In the infrared spectra, the characteristic C-N stretching band of the triazine units was observed at 1384 and 1508 cm^−1^, which slightly shifted from the C-N breathing and stretching mode of a molecular triazine unit (1363 and 1511 cm^−1^, respectively), in agreement with the infrared spectra of other CTFs [3,36]. At the same time, the CN band of the monomer at 2227 cm^−1^ disappeared (Figure S2, Supplementary Materials), signaling that the monomer was consumed during polymerization.

The CHN elemental combustion analysis reveals the typical nitrogen loss which increases with a higher temperature (Table S1). Nitrogen loss is due to a partial aromatic nitrile decomposition into HCN, CN radicals, NH_3_, and other species from the synthesis at temperatures of several hundred °C [3,5,35,37,38,39,40,41,42]. It can be seen that the C/N ratio is increased while the C/H ratio does not change much and stays close to the theoretical ratio, thereby indicating the primary loss of nitrogen-rich species. These results correlate with the general observation that an increase in temperature in ionothermal CTF synthesis leads to enhanced carbonization of the samples [5,12,39]. To avoid nitrogen loss, we also performed pBN-CTF formation at 350 °C. It became evident, however, that the surface area, porosity, and gas sorption of the samples at 350 °C varied greatly from batch to batch and among different probes from the same batch (Section S4, Figures S5 and S6, Table S2). The surface area of the pBN-CTF-10-350, which is based on N_2_ gas adsorption, ranged from 660 to 1027 m^2^ g^−1^ across three batches (Figure S5a). CO_2_ adsorption also showed variation across the three different batches and in addition across three probes from the same batches, with the uptake varying from 38 to 66 cm^3^ g^−1^ (Figure S6).

Thus, the samples at 350 °C were inhomogeneous and could not be reproducibly synthesized. Therefore, the results from the reaction temperature at 350 °C were not included in the discussion here in the main text.

The remaining difference in the combined weight percentage of C, H, and N to 100% amounts to ~20% and is usually explained by residual ZnCl_2_ or by the adsorption of water upon sample handling. In the literature, it is well known and frequently stated that the ionothermal ZnCl_2_ route gives hard-to-remove ZnCl_2_ metal impurities from the needed 5–10 times molar excess [8,21]. Energy-dispersive X-ray spectroscopy (EDX) gave a consistent amount of both Zn (~4.5 wt%) and Cl (7–10 wt%, Table S1). ZnCl_2_, which is embedded in the pores of the CTFs, is difficult to remove even by extended washing, as some of the pores may no longer be accessible. The still remaining difference of ~5–15 wt% was shown to be due to the adsorption of moisture from air in the porous CTF. We have recently verified that CTFs are hygroscopic, with a water uptake of up to 0.12–0.20 g g^−1^ (equivalent to 11–17 wt%) at 50–60% air humidity (that is P/P_0_ ≈ 0.5–0.6) when handled or stored under ambient air [14,39,43].

Powder X-ray diffractograms (PXRDs) yield only broad reflexes without any clear signature of (001) reflections for parallel two-dimensional sheets in eclipsed stacking (Figure S3), which indicates a very amorphous structure because of defects in the idealized hexagonal sheets with possibly partial interpenetration or three-dimensional framework arrangements.

**Figure 2:**
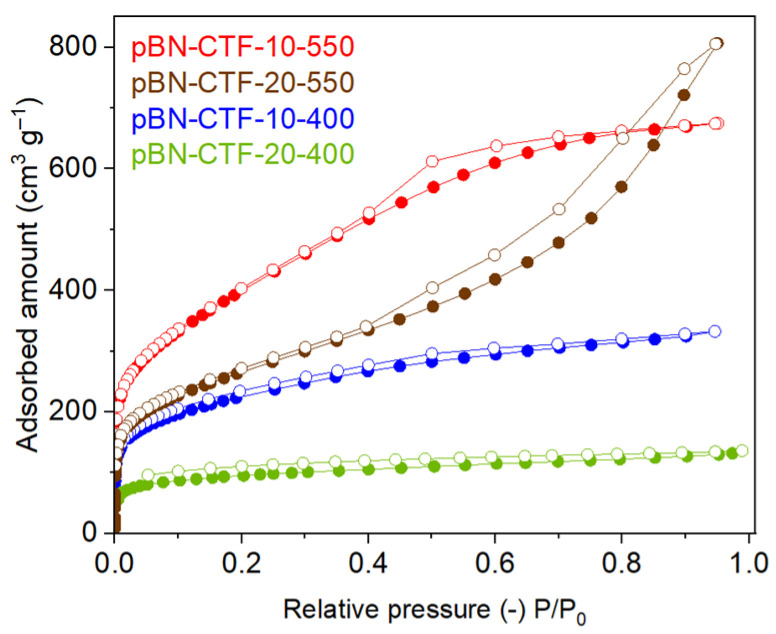
N_2_ isotherms (at 77 K) of pBN-CTFs (filled symbols adsorption, empty symbols desorption).

**Figure 3:**
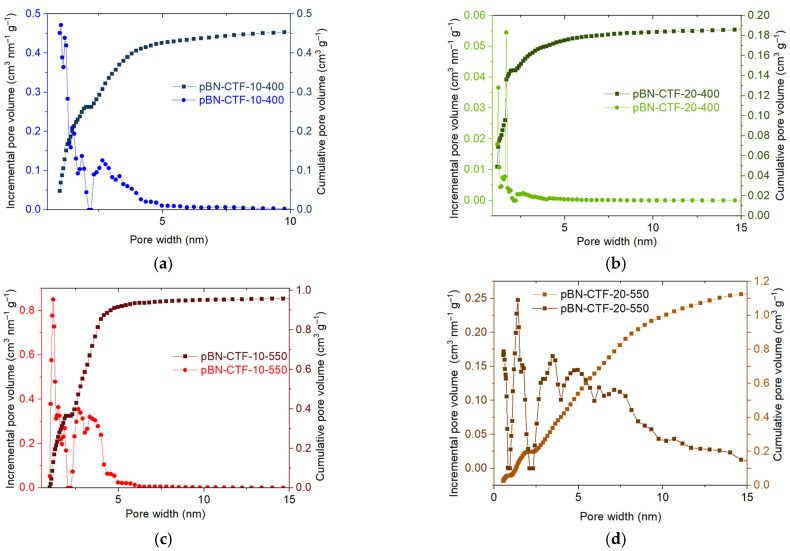
NLDFT pore size distribution (PSD) curves showing the cumulative pore volume (right y axes) and the incremental pore volume (left y axes) of pBN-CTF from N_2_ adsorption using the “N_2_ at 77 K on carbon slit pore, NLDFT equilibrium model” for (**a**) pBN-CTF-10-400, (**b**) pBN-CTF-20-400, (**c**) pBN-CTF-10-550, and (**d**) pBN-CTF-20-550.

The nitrogen sorption isotherms of the CTFs in Figure 2 all show a pronounced adsorption step at P/P_0_ < 0.05 corresponding to gas sorption in the micropores (pores < 2 nm, Figure 3, see Figure S5b for the 350 °C samples). The adsorption isotherms at 400 °C are largely of type Ib, indicative of materials with micropore size distributions over a broader range and narrow mesopores (pores > 2 nm, Figure 3a,b, Table 2) [1]. There is an H4 hysteresis, where the hysteresis loop closes only at very low relative pressure P/P_0_. Such H4 loops are found among others with micro-mesoporous carbons [1,2]. For 550 °C, the adsorption isotherm of the 10–550 sample appears to be a Type I and IV combination. The adsorption branch has a “knee” at P/P_0_ ~ 0.4 and the saturation plateau, which is a typical feature of Type IV isotherms, is then reached at high P/P_0_. Type IV isotherms are given by largely mesoporous adsorbents (Figure 3c,d, Table 2). The isotherm at 550 °C has a hysteresis loop of Type H2b, which is associated with pore blocking in a wide range of pore neck widths. The N_2_ adsorption isotherm of the sample 20–550 can be assigned as a mixture of Type I and Type II isotherms. The nitrogen uptake does not saturate towards P/P_0_ = 1, which is due to a Type II branch. Type II indicates macropores (pores > 50 nm), which can also be caused by the voids between the particles. The isotherm has an H3 hysteresis loop that correlates with macropores that are not filled with pore condensate [1,2].

The specific surface areas were obtained from the Brunauer–Emmett–Teller (BET) model over the pressure range of P/P_0_ ≈ 0.01–0.07. Generally, the surface areas are higher for the 10:1 than for the 20:1 ZnCl_2_/monomer ratios, giving 809–1460 m^2^ g^−1^ for the former and only 348–950 m^2^ g^−1^ for the latter (Table 2).

In other CTF synthesis, e.g., with the tetra(4-cyanophenyl)ethylene monomer, ZnCl_2_/monomer ratios of 10:1 and 20:1 were compared, with the former giving a more than two-fold higher surface area (2235 vs. 784 m^2^ g^−1^) [37,44]. Thus, a ratio of 10:1 seems optimal for many ionothermal CTF syntheses. In the following, we will therefore only discuss the results for the 10:1 molar ratio; that is, the pBN-CTF-10 series. In agreement with other CTF works, the surface area of the sample synthesized at 400 °C is lower than that at 550 °C (Table 2, Figure 2) as generally the surface areas and total pore volumes for the resulting products increase with temperature [39]. The surface areas of the pBN-CTFs are comparable with other CTFs with longer linkers, e.g., terphenyl prepared by Kuhn et al. with a surface area of 975 m^2^ g^−1^ [21], or even 2,8-dicyano-6H,12H-5,11-methanodibenzo [1,5]diazocine that was synthesized by Wang et al. with 612 m^2^ g^−1^ [45].

Using NL-DFT calculations with a slit pore model on the N_2_ adsorption isotherms, the pore widths and distribution as well as the total and micropore volume can be estimated (Figure 3 and Figure S5, Table 2) [35]. We can note that for their amorphous nature, as evidenced by PXRD (Figure S3), the pBN-CTF-10-400, -20-400, and -10-550 materials feature a surprisingly narrow pore size distribution within 1–5 nm for 90% of the total pore volume (Figure 3a–c). The pore width distribution diagrams for the CTF-400s indicate pronounced maxima in the micropore region (<2 nm) and pore sizes larger than 2 nm up to 5 nm in a broad distribution. At 400 °C, the micropore volume encompasses more than 50% of the total pore volume; that is, the V_micro_/V_tot_ values are above 0.50 (Table 2). At 550 °C, the total pore volume more than doubles in comparison to 400 °C, and the pore width distributions exhibit a broader contribution of mesopores between 2 and ~5 nm (Figure 3c) and beyond (Figure 3d), such that V_micro_/V_tot_ drops below 35% (Table 1). Notably, the material with the highest surface area, namely pBN-CTF-10-550, has the lowest micropore volume fraction V_micro_/V_tot_ among all the pBN-CTF materials listed in Table 2.

**Figure 4:**
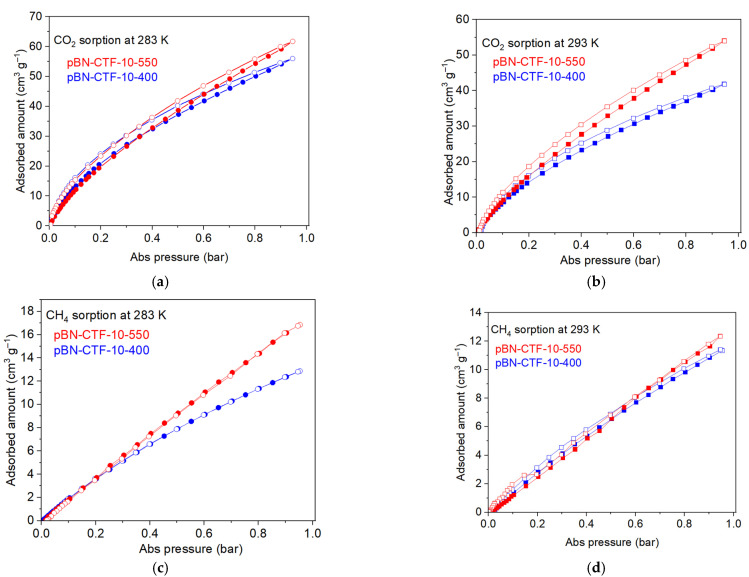
Adsorption and desorption isotherms of pBN-CTF-10-400 and pBN-CTF-10-550 for (**a**) CO_2_ at 283 K, (**b**) CO_2_ at 293 K, (**c**) CH_4_ at 283 K, and (**d**) CH_4_ at 293 K (filled symbols adsorption, empty symbols desorption). The CO_2_ adsorption isotherms at 195 K are given in Figure S7a, and those for pBN-CTF-20-400 and -550 at 293 K are given in Figure S8.

The pore size distribution (PSD) from N_2_ sorption at 77 K is generally limited to pores between ~1 and ~40 nm. Macropores (>50 nm) are not accounted anymore by N_2_ sorption. For pores smaller than 1 nm (10 Å), the size and distribution need to be obtained from CO_2_ gas adsorption data, because for N_2_ sorption at 77 K, the diffusion of the molecules into micropores smaller than 1 nm is very slow; hence, it requires very long N_2_ adsorption measurements for equilibration of the adsorption isotherms, which cannot be assured. To avoid erroneous PSD results from the N_2_ adsorption analysis, CO_2_ adsorption analysis can be used (Figure 4a,b). The saturation pressure of CO_2_ at 10 °C is ~4480 kPa (~33,450 Torr), so that a low relative pressure, which is necessary for the micropore analysis, is achieved in the range of moderate absolute pressures [46]. The micropore analysis with CO_2_ at 283 K instead of N_2_ at 77 K allows for a faster equilibration and access of even smaller pores as the kinetic diameter of CO_2_ is only 3.30 Å versus 3.64 Å for N_2_. The NL-DFT analysis of the CO_2_ adsorption isotherms of the pBN-CTF-10s with the “CO_2_ on carbon-based slit pore” model yield similar corrugated pore size distribution curves for the CTFs below 1 nm with pronounced maxima between 0.5 and 0.9 nm and at ~0.85 nm (Figure 2). The surface area was also calculated using the CO_2_ adsorption at 195 K (values can be seen in Table 3 and isotherms in Figure S7a), which gave a smaller surface area than that of the N_2_ counterpart, corresponding to the literature [37,47,48,49,50].

Volumetric CO_2_ and CH_4_ adsorption studies resulted in the isotherms depicted in Figure 4. At 283 K and 293 K, the pBN-CTF-10 materials show similar CO_2_ sorption isotherm curvatures that did not level off much at 1 bar but still have a rather positive slope, which indicates that the uptake at 1 bar is far from saturated. At 195 K, the CO_2_ uptake at 1 bar differentiates considerably for the pBN-CTF-10-400 and 10-550 material (Table 3, Figure S7), increasing nearly two-fold, from pBN-CTF-10-400 with 175 cm^3^ g^−1^ to pBN-CTF-10-550 with 320 cm^3^ g^−1^. As shown in Figures S9 and S10, this increase correlates with the increase in surface area and pore volume from the 400 °C to the 550 °C material in Table 2.

**Figure 5:**
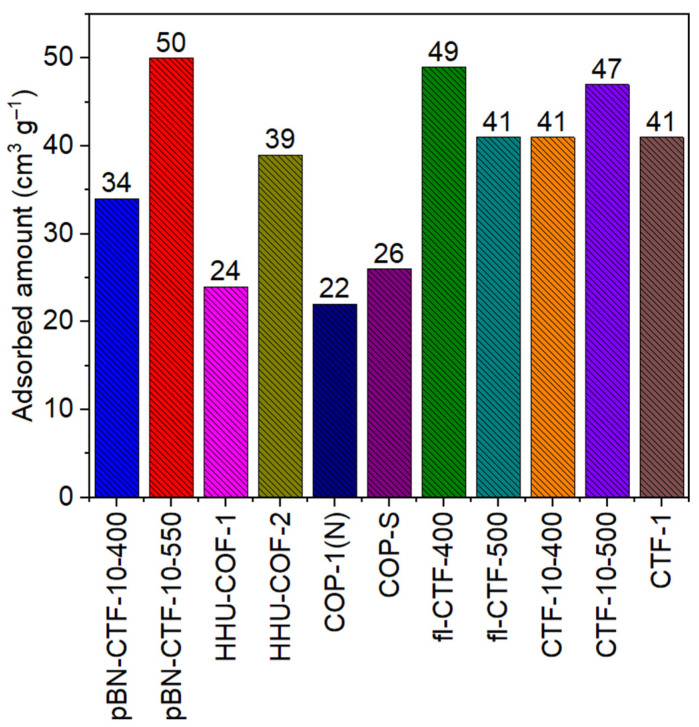
CO_2_ uptake comparison at 298 K and 1 bar between the pBN-CTFs and selected CTFs with data from Table S4 [17,49,51,52,53]. The CTFs all have a linker with a length of at least a biphenyl unit, except for the reference of prototypical CTF-1. HHU-COF-1 is based on the monomer [1,1′-biphenyl]-4,4′-dicarbaldehyde, HHU-COF-2 on 2,2′,3,3′,5,5′,6,6′-octafluoro-[1,1′-biphenyl]-4,4′-dicarbaldehyde, condensed both with 1,3,5-tris-(4-aminophenyl)triazine [17]. COP-1(N) and COP-S are mixed linker CTFs from 2,4,6-trichloro-1,3,5-triazine with piperazine and 2,7-diazaspiro-[4,4]-nonane and [48], fl-CTF-400 and -500 are based on the 9H-fluorene-2,7-dicarbonitrile monomer [51], CTF-10-400 and -500 on 4,4′,4″,4‴-(1,4-phenylenebis(pyridine-4,2,6-triyl))tetrabenzonitrile [52], and CTF-1 on terephthalonitrile [53] (see Scheme S1 for the monomer structures). Table S7 summarizes the BET surface area, CO_2_ uptake capacity, Q_ads_, and CO_2_/CH_4_ IAST selectivity of the CTFs given here in Figure 5.

By comparing the pBN-CTFs from this work to other CTFs with linkers equal or longer than a biphenyl unit, it can be seen that the pBN-CTFs can compete very well in terms of CO_2_ uptake (Figure 5, Table S4).

Covalent triazine frameworks are widely investigated for CO_2_/N_2_ and CO_2_/CH_4_ separation. The ideal adsorbed solution theory (IAST) can give an indication of the selectivity of different gas mixtures at a given pressure or for a given gas mixture at different pressures. The only criterion that IAST requires is that both gases should have an equal spreading pressure at the given temperature [54]. The IAST selectivity is derived from the single gas adsorption isotherms and was calculated here on the bases of fitting the adsorption isotherms with the Freundlich–Langmuir adsorption model, and the parameters that resulted from the isotherm fitting (Table S5) were used to calculate the selectivity (Table 3). For example, the sample pBN-CTF-10-400 at 293 K has a maximal loading of 7.8 mmol g^−1^ and 1.8 mmol g^−1^ for CO_2_ and CH_4_, respectively, and an affinity constant of 0.32 mmol g^−1^ bar^−1^ and 0.41 mmol g^−1^ bar^−1^ for CO_2_ and CH_4_, respectively, with an R^2^ value of 0.999 for both fits (Table S5).

IAST underscores the selectivity for CO_2_ over CH_4_ for the pBN-CTF-10-400 material, as seen already in the higher uptake of CO_2_ over CH_4_ at the same temperature (Table 3). At 283 K, the slight pressure and composition dependent CO_2_/CH_4_ selectivity for pBN-CTF-10-440 varies between 7 and 22. It decreases with pressure and increases with an increasing CH_4_ fraction (Figure S13). At 293 K, the CO_2_/CH_4_ selectivity for pBN-CTF-10-440 stays rather constant between 0.01 and 0.8 CH_4_ molar fraction. The preference for CO_2_ can be explained from the pore structure and the interaction strength between the gas molecules and the framework. pBN-CTF-10-400 has good microporosity and nitrogen content. Micropores favor CO_2_ adsorption due to its smaller kinetic diameter (3.3 Å) compared to CH_4_ (3.8 Å), while nitrogen functionalities enhance CO_2_ affinity through dipole-quadrupole interactions.

**Figure 6:**
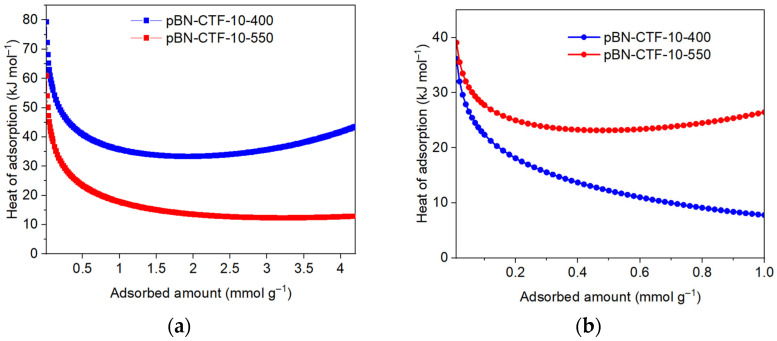
Isosteric heat of adsorption for (**a**) CO_2_ and (**b**) CH_4_ on pBN-CTF-10-400 and 550.

From the measurement of gas adsorption at two temperatures with ∆T = 10 to 20 °C, the enthalpy (∆H) or heat of gas adsorption (Q_ads_ = −∆H) can be obtained [15,55]. Near zero coverage, the heat of adsorption for CO_2_ is remarkably high in comparison to other CTFs, with 79 kJ mol^−1^ for pBN-CTF-10-400 (Table S6). In Figure 6, the isosteric heat of adsorption was plotted against the amount of CO_2_ and CH_4_ adsorbed by the frameworks. pBN-CTF-10-400 has a higher microporosity and higher nitrogen content than pBN-CTF-10-550. This relates to a higher affinity for CO_2_ than for CH_4_ because (as just noted) CO_2_, with its smaller kinetic diameter (3.3 Å) compared to CH_4_ (3.8 Å), can occupy smaller micropores, and the CO_2_ quadrupole can interact with the dipole of nitrogen functionalities. Micropores generally allow for multi-site or “wall–guest–wall” interactions between guest molecules and the inner pore surface [56]. The large decrease in the isosteric heat of adsorption from a near zero adsorbed amount to ~0.5 mmol g^−1^ adsorbed amount seen in Figure 6 for both gases is due to the initial filling of the very small or ultra-micropores with a diameter in the dimension of the adsorbate molecule with wall-to-wall interactions and the occupation of the nitrogen atom sites, which also have higher adsorption energies. Notably, the CO_2_ heat of adsorption values of pBN-CTF-10-550 drop below the liquefaction heat of CO_2_ of 17 kJ mol^−1^ when the adsorbed amount surpasses 1.1 mmol g^−1^, while the heat of adsorption of pBN-CTF-10-400 stays well above the liquefaction heat of CO_2_. A drop below the heat of liquefaction of CO_2_ indicates weaker adsorbate–surface interactions than adsorbate–adsorbate interactions in the liquid phase. This behavior is beneficial in pressure or temperature swing adsorption (PSA/TSA) applications as it facilitates easier desorption with a lower energy input.

Conversely, pBN-CTF-10-550 exhibits a higher Q_ads_ for CH_4_ over the whole uptake range compared to pBN-CTF-10-400 and also a higher heat of adsorption for CH_4_ than for CO_2_ once the adsorbed CO_2_ amount exceeded ~0.4 mmol g^−1^ (compare Figure 6a,b). This can be explained by the more carbon-like non-polar nature of the pBN-CTF-10-550 material with less nitrogen content than pBN-CTF-10-400, which gives the former a relatively higher affinity to non-polar CH_4_ [57,58]. As expected, the heat of adsorption near zero coverage is lower for CH_4_ than for CO_2_ for both CTFs (Table 3). A further comparison of the literature for CO_2_ uptake and Q_ads_^0^ for CO_2_ in CTFs can be found in Table S4 and Table S7, respectively, in the Supplementary Materials [14,18,25,26,59,60,61,62,63,64,65,66,67,68,69].

The thermogravimetric analysis showed that under air, all samples started decomposing (with weight loss) at ~400 °C, including the pBN-CTF, which was synthesized at 550 °C (Figure S14). This mass loss of the pBN-CTF-10-400 sample is complete below 700 °C with a residual mass of ~2.5 wt%. The mass loss of pBN-CTF-10-550 continues to ~770 °C, leaving only ~0.2 wt%.

## 3. Materials and Methods

### 3.1. Instrumentation

Fourier transform infrared spectroscopic measurements were taken using a Bruker Tensor 37 (Bruker AXS, Karlsruhe, Germany) with KBr pellets in the range between 4000 and 500 cm^−1^. For the N_2_ sorption analysis, a Quantachrome Autosorb-IQ-MP (Quantachrome, Boynton Beach, FL, USA) was used. The samples were degassed for 24 h at 120 °C before connecting to the device. The measurement was taken at 77 K. The results were interpreted with the BET equation. The CO_2_ sorption analysis was performed with a Quantachrome Autosorb-IQ-MP (Quantachrome, Boynton Beach, FL, USA). The measurement temperatures were 293, 283, and 195 K after activating (degassing) the samples under vacuum at 120 °C for 24 h. The temperature was held by virtue of a thermostated water bath (293 and 283 K) or with a cryodyne refrigerator model 8200 (195 K) (Janis, Woburn, MA, USA).

Thermogravimetric analysis was performed with a TG Tarsus 209 F3 (Netzsch, Selb, Germany). The samples were analyzed under synthetic air with a heating rate of 10 K/min from 25 to 900 °C. Powder X-ray diffraction patterns were recorded using a Bruker D2 phaser from Bruker (Bruker AXS, Karlsruhe, Germany) with Cu-Kα radiation, λ = 1.54182 Ǻ at 300 W, 30 kV, 10 mA. Nuclear magnetic resonance (^1^H-NMR) spectra were collected with a Bruker Avance III-600-I (Bruker, Karlsruhe, Germany). The chemical shifts are given in ppm and are referenced to the residual proton signal of the deuterated solvent (7.26 ppm for CDCl_3_, 7.16 for C_6_D_6_).

### 3.2. Chemicals

Phenazine (99.86%) and 4-bromobenzonitrile (95%) were obtained from BLDpharm (Reinbek, Germany). Sodium dithionite (85%) was obtained from VWR chemicals (Darmstadt, Germany). Palladium acetate (99.9%) and tri-tert-butyl phosphine (99%) were purchased from Sigma-Aldrich (Darmstadt, Germany). All solvents were purchased from commercial suppliers with a minimum purity of 99.8%.

### 3.3. Synthesis of 5,10-Dihydrophenazine

Following the literature [70], a phenazine (2.5 g, 13.87 mmol) solution in ethanol (30 mL) and a sodium dithionite (24.1 g, 137 mmol) solution in water (125 mL) were placed into a round-bottom flask and heated to reflux at 95 °C for 3 h. Afterwards, the flask was cooled to room temperature and then the product was separated by filtration, washed three times with water (3 × 15 mL), dried under vacuum (10^−3^ mbar), and stored under nitrogen to avoid any oxidation. The yield was 2.10 g, 85%.

^1^H NMR (600 MHz, CDCl_3_) δ 8.27 (dd, *J* = 6.8, 3.5 Hz, 1H), 7.86 (dd, *J* = 6.8, 3.4 Hz, 1H), 6.12 (s, 2H), 1.57 (s, 3H).

### 3.4. Synthesis of 4,4′-(Phenazine-5,10-diyl)dibenzonitrile (pBN)

5,10-Dihydrophenazine (2 g, 11 mmoL), 4-bromobenzonitrile (4.38 g, 24 mmoL), and potassium carbonate (9.1 g, 65.8 mmoL) were combined in degassed toluene (80 mL) in a round-bottom flask under N_2_ atmosphere according to the literature [70]. To this mixture, palladium acetate (0.141 g, 0.62 mmoL) and tri-tert-butyl phosphine (0.464 g, 2.30 mmoL) dissolved in 10 mL of toluene were added; the flask was then refluxed at 111 °C for 20 h. During cooling, water (30 mL) was added to the reaction mixture in order to stop the reaction. The product was extracted from the water phase using chloroform (200 mL). The separated organic phase was washed with brine (saturated aqueous NaCl solution) three times (3 × 30 mL) and was dried over magnesium sulfate (MgSO_4_) for 15 min. The organic phase was then filtered and concentrated via a rotary evaporator to ~50 mL; after that, 100 mL of n-hexane was added and cooled in an ice bath for 10 min. The separated product was filtered and dried in a vacuum oven (10^−3^ mbar) at 60 °C.

^1^H NMR (600 MHz, C_6_D_6_) δ 6.96 (d, *J* = 8.2 Hz, 4H), 6.71 (d, *J* = 8.2 Hz, 4H), 6.41–6.37 (m, 4H), 5.69 (dt, *J* = 7.9, 3.9 Hz, 4H).

### 3.5. pBN-CTF Synthesis

Inside the glove box, a glass ampule with a Schlenk fitting was filled with (0.2 g, 0.5 mmol) pBN-2CN and 10 or 20 equivalents (0.680 g, 5 mmol or 1.3 g, 10 mmol) of anhydrous zinc chloride. Outside the glovebox, the ampule was evacuated and flame-sealed and heated for 48 h in a tube furnace at the chosen temperature of 350, 400, or 550 °C. The 350 and 400 °C reactions were carried out in a normal borosilicate glass (Pyrex); for 550 °C, a quartz glass ampule was used. After cooling the ampule to room temperature, the ampule was carefully opened with no sign of pressure built-up inside.

The reaction product was stirred in distilled water acidified with 0.5 mol/L of hydrochloric acid (HCl) to pH = 4 (50 mL) for three days. The stirring was vigorous to ensure the mechanical break-up of the black monolith to provide fine particles. After additional stirring for 72 h in distilled water, the product was filtered and washed with the organic solvents chloroform, acetone, and methanol (30 mL each) in this order. After the washing, the product was dried in a vacuum (10^−3^ mbar) oven at 60 °C for 24 h. The yields are listed in Table 1.

## 4. Conclusions

The molecule 4,4′-(phenazine-5,10-diyl)dibenzonitrile, with a long—about 12 Å—separation between the nitrile groups, can be successfully transformed by ionothermal synthesis into a porous covalent triazine framework (pBN-CTF). The surface area increases expectedly with the synthesis temperature and ranges from 809 to 1460 m^2^ g^−1^ for the samples synthesized at 400 and 550 °C, respectively, with pores ranging between 1 and 4 nm. The pBN-CTF exhibited a good CO_2_ uptake at 293 K, showing a similar performance to benchmark materials like CTF-1, due to the relatively high micropore fraction that ranged between 35% for the 550 °C and 50% for the 400° samples. The new material showed a significant difference and stark contrast to the adsorption of CO_2_ over CH_4_ for the potential separation, with a selectivity that reaches 22. For further work on pBN-CTFs and other CTFs in general, we plan to increase the nitrogen content of the formed framework through the addition of a nitrogen-rich compound such as melamine in order to introduce additional electron pair donors and thereby influence the adsorption properties. We will also check the elongation of the phenyl group in pBN with a biphenyl group, giving the monomer 4′,4‴-(phenazine-5,10-diyl)bis(([1,1′-biphenyl]-4-carbonitrile)) that can be assumed to form CTFs with an even larger pore width of over 2 nm channel cross-sections to allow for faster mass transport; that is, diffusion through the then hierarchical micro-mesopores.

## Figures and Tables

**Table 1:** Summary of the reaction parameters and yields for pBN-CTFs.

CTF Product ^(a)^	Molar Ratio ZnCl_2_/Monomer	Temperature (°C)	Yield (%)
pBN-CTF-10-350	10	350	79
pBN-CTF-20-350	20	350	78
pBN-CTF-10-400	10	400	68
pBN-CTF-20-400	20	400	92
pBN-CTF-10-550	10	550	84
pBN-CTF-20-550	20	550	40

^(a)^ The first number in the product name after CTF gives the molar ZnCl_2_/monomer ratio (10 or 20), followed by the reaction temperature (400 or 550 °C).

**Table 2:** Surface area and porosity data from N_2_ and CO_2_ sorption studies.

CTF Product	S_BET_ ^(a)^ (m^2^ g^−1^)	V_tot_ ^(b)^ (cm^3^ g^−1^)	V_micro_ ^(c)^ (cm^3^ g^−1^)	V_micro_/V_tot_^(d)^	V_1nm_(CO_2_) ^(e)^ (cm^3^ g^−1^)
pBN-CTF-10-400	809	0.51	0.25	0.50	0.015
pBN-CTF-20-400	348	0.19	0.15	0.79	0.009
pBN-CTF-10-550	1460	1.04	0.36	0.35	0.013
pBN-CTF-20-550	950	1.25	0.19	0.31	0.010

^(a)^ Calculated BET surface area from N_2_ adsorption at 77 K over a pressure range of P/P_0_ = 0.01–0.07. ^(b)^ Total pore volume from N_2_ adsorption isotherm at 77 K at P/P_0_ = 0.95 for pores smaller than 40 nm. ^(c)^ Micropore volume from the NL-DFT method using the N_2_ adsorption isotherm at 77 K at P/P_0_ = 0.1 for pores with d ≤ 2 nm (20 Å). ^(d)^ Micropore volume/total pore volume. ^(e)^ Pore volume for pores with diameters smaller than 1 nm from CO_2_ adsorption isotherms at 293 K and the CO_2_ NL-DFT model.

**Table 3:** CO_2_ and CH_4_ adsorption results at 1 bar and heat of adsorption for CO_2_ at zero coverage and CO_2_:CH_4_ selectivity.

CTF Product	S_BET_ (195 K) ^(a)^ (m^2^ g^−1^)	CO_2_ (cm^3^ g^−1^)			CH_4_ (cm^3^ g^−1^)		CO_2_ Q_ads_^0 (b)^ (kJ mol^−1^)	CH_4_ Q_ads_^0 (b)^ (kJ mol^−1^)	IAST Selectivity for 50:50 CO_2_:CH_4_
		293 K	283 K	195 K	293 K	283 K			
pBN-CTF-10-400	524	42.8	55.9	175	11.3	12.9	79	36	22
pBN-CTF-10-550	746	54.0	61.7	320	12.3	16.9	60	39	- ^(c)^

^(a)^ BET surface area from CO_2_ adsorption measured at 195 K in the range between 0.08 and 0.2 P/P_0_. The difference in surface area between N_2_ (77 K) and CO_2_ (195 K) can be due to the kinetic energy difference at different temperatures and also the size of the molecules adsorbed. ^(b)^ Isosteric heat of adsorption of CO_2_ or CH_4_ towards zero loadings from the adsorption isotherms at 283 K and 293 K. ^(c)^ IAST selectivity for 50:50 mol:mol or equimolar fraction of CO_2_ and CH_4_ at 293 K and 1 bar. The linear CH_4_ uptake of CTF-10-550 did not allow for a meaningful fit.

## Data Availability

The original contributions presented in this study are included in the article/Supplementary Materials. Further inquiries can be directed to the corresponding author.

## Supplementary Materials

The following supporting information can be downloaded at: https://www.mdpi.com/article/10.3390/molecules30153110/s1, Section S1. Scanning electron microscopy; Section S2. Fourier transform infrared spectroscopy and elemental analysis; Section S3. Powder X-ray diffraction; Section S4. Samples synthesized at 350 °C and their N_2_ and CO_2_ sorption studies; Section S5. CO_2_ adsorption isotherms; Section S6. Calculations and fitting for the isosteric heat of adsorption and IAST selectivity of CO_2_ and CH_4_; Section S7. Thermogravimetric analysis (TGA); Section S8. Nuclear magnetic resonance spectrometry (NMR); Section S9. References.
