# Supplementary material for: Synthesis and Characterization of Covalent Triazine Frameworks Based on 4,4′-(Phenazine-5,10-diyl)dibenzonitrile and Its Application in CO2/CH4 Separation
Source: Molecules. 2025 Jul 24;30(15):3110. doi: 10.3390/molecules30153110 (PMC12348490; doi:10.3390/molecules30153110)
Supplement: Supplementary file 1 [file molecules-30-03110-s001.zip › molecules-3762622-supplementary.pdf]

Supplementary Materials:

Synthesis and Characterization of Covalent Triazine Frameworks Based on 4,4'-(Phenazine-5,10-diyl)dibenzonitrile and Its Application in CO_2/CH_4 Separation

Hanibal Othman, Robert Oestreich, Vivian Küll, Marcus N. A. Fetzer and Christoph Janiak *

Institut für Anorganische Chemie und Strukturchemie, Heinrich-Heine-Universität Düsseldorf, 40204 Düsseldorf, Germany; hanibal.othman@hhu.de (H.O.); robert.oestreich@hhu.de (R.O.); vivian.kuell@hhu.de (V.K.); marcus.fetzer@hhu.de (M.N.A.F.)

* Correspondence: janiak@uni-duesseldorf.de

Section S1. Scanning electron microscopy

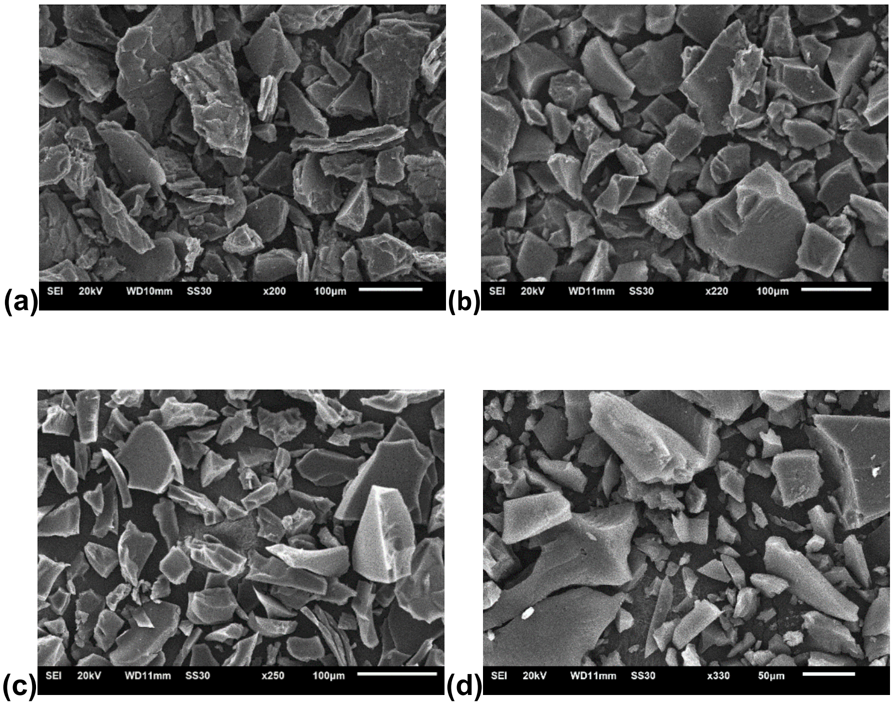

Figure S1. SEM images of (a) pBN CTF-10-400, (b) pBN -CTF-20-400, (c) pBN-CTF-10-550 and (d) pBN-CTF-20-550.

Section S2. Fourier transform infrared spectroscopy and elemental analysis

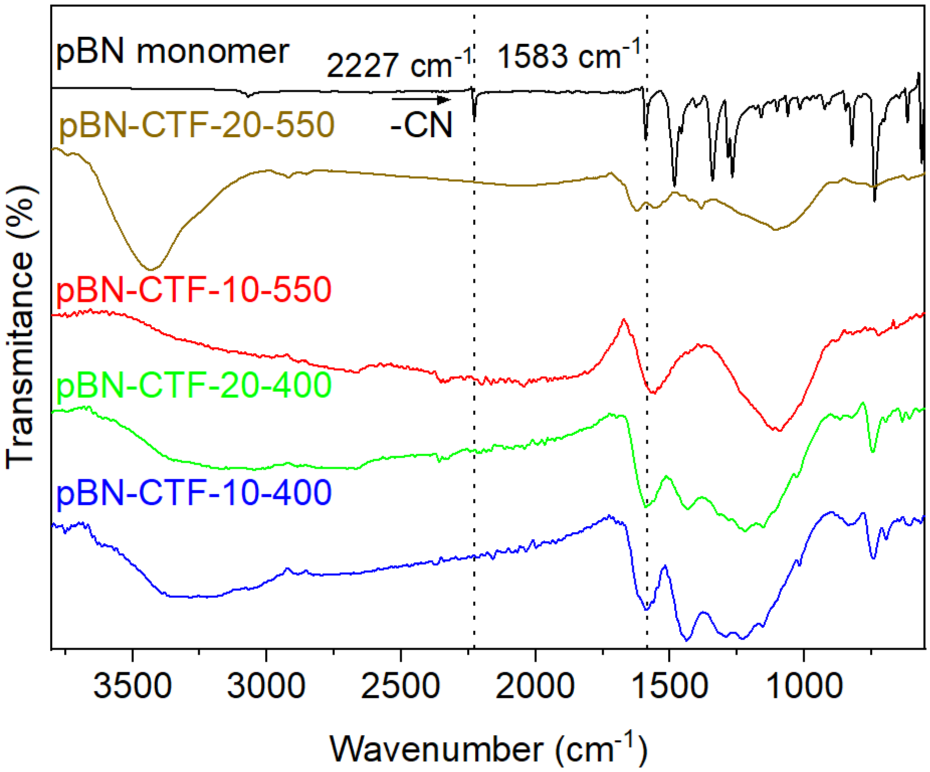

Figure S2. FT-IR spectra of the synthesized pBN-CTFs and the pBN monomer (in black).

Elemental analysis:

Table S1. Elemental analysis results.[(a)]

CTF product	C (wt%)	H (wt%)	N (wt%)	residual wt%	C/N mass ratio	C/H mass ratio
**Theoretical CTF**	81.2	4.2	14.6	0.00	5.5	19.4
**pBN-CTF-10-350**	68.5	4.2	7.9	19.4	8.6	16.3
**pBN-CTF-10-400**	68.3	3.5	6.5	21.7	10.5	19.5
**pBN-CTF-10-550**	60.7	3.3	5.2	30.8	11.6	18.4
**pBN-CTF-20-400**	69.3	3.4	6.4	20.9	10.6	20.4
**pBN-CTF-20-550**	61.6	3.2	5.0	30.2	12.3	19.3

[(a)] From combustion analysis.

CTF product	Zn (wt%) [(b)]	Cl (wt%) [(b)]	Sum Zn+Cl (wt%) [b]
**pBN-CTF-10-350**	4.3	9.8	14.1
**pBN-CTF-10-400**	4.7	8.6	13.3
**pBN-CTF-10-550**	4.5	9.5	14.0
**pBN-CTF-20-400**	4.4	8.1	12.5
**pBN-CTF-20-550**	4.2	7.0	11.2

[(b)] From SEM-EDX

Section S3. Powder X-ray diffraction

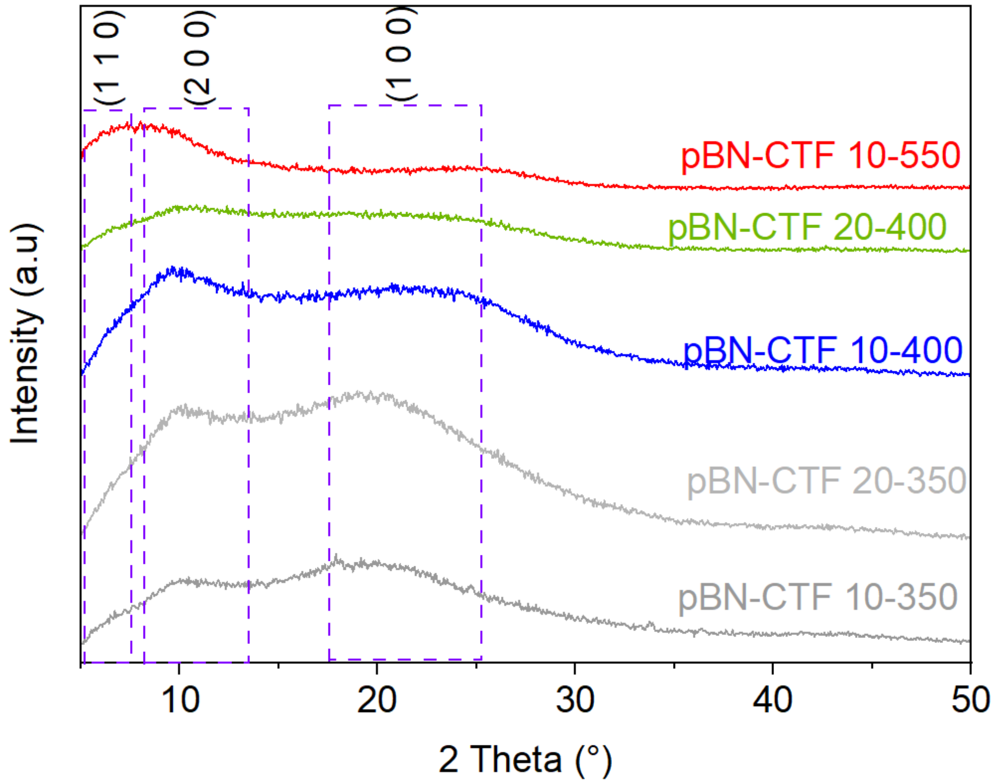

Figure S3. Powder X-ray diffraction patterns of pBN-CTFs. The regions for the (1 1 0), (2 0 0) and (1 0 0) reflections which are expected in more crystalline pBN-CTFs are indicated, based on the estimation from the hexagon edge and width lengths in Figure S4. 2Theta for the reflection from the hexagonal layers in eclipsed stacking with a separation of 3.5 Å (0 0 1 reflection) is 25.4°.

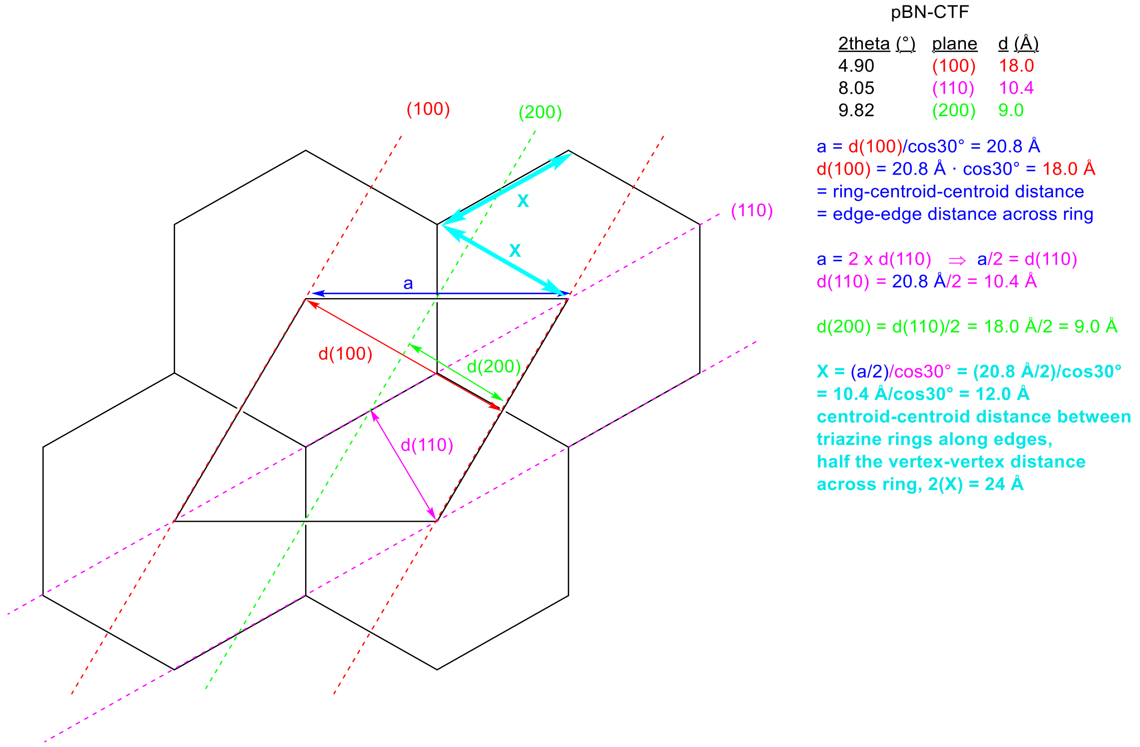

Figure S4. Correlation of the estimated edge length (X = 12.0 Å) and width (a = 20.8 Å) of the ideal hexagon (cf. Figure 1 in the main text) with the 2theta (2θ) values expected in the powder X-ray diffractograms in Figure S3 from the reflection planes and the d spacing according to the Bragg equation n λ = 2d sinθ or d = nλ /(2sinθ) with λ = 1.5406 Å and n = 1 [1].

Section S4. Samples synthesized at 350 °C and their N_2 and CO_2 sorption studies

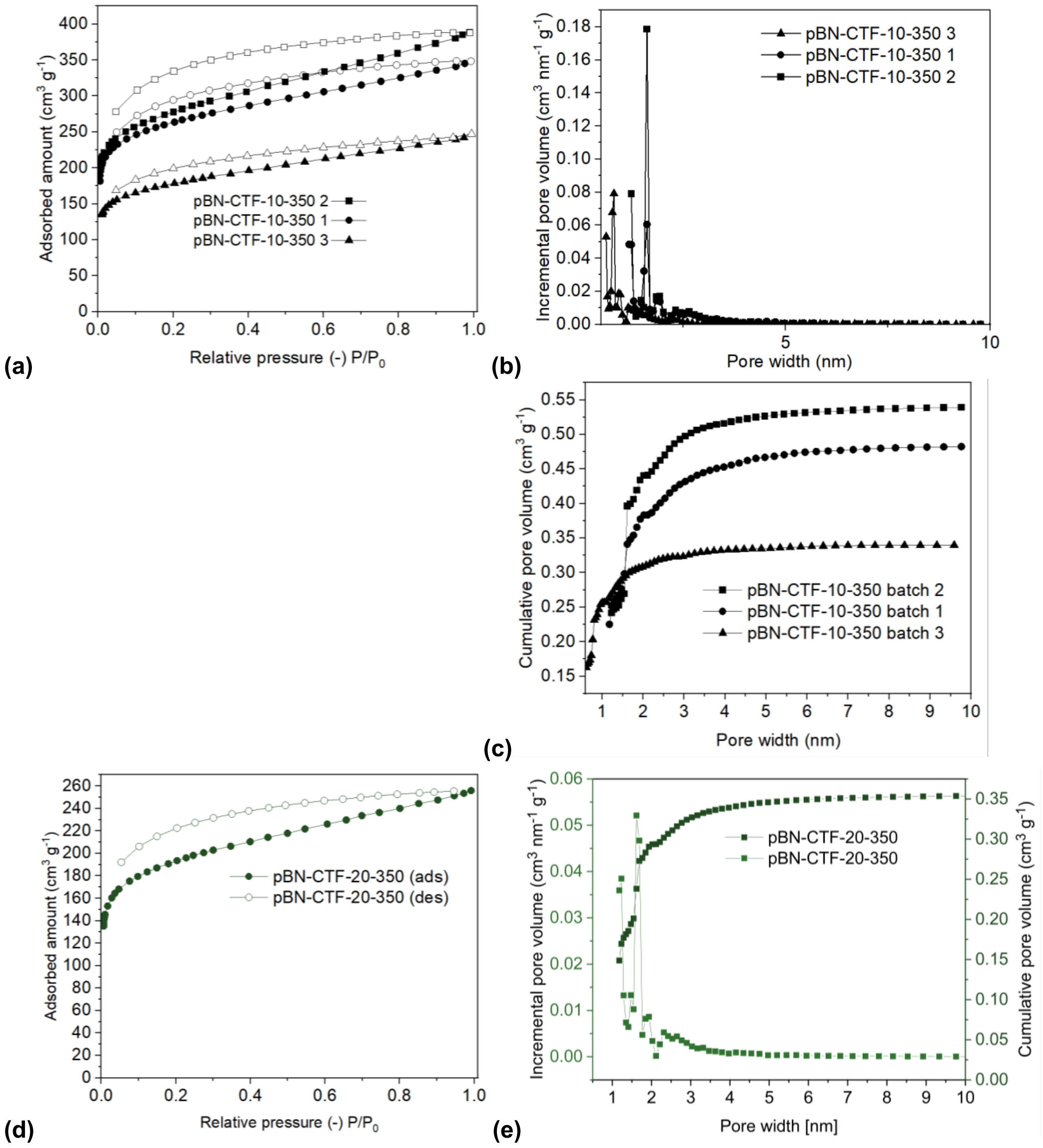

Figure S5. (**a**) N_2 sorption isotherms at 77 K of the samples synthesized with a 10:1 $ZnCl_2$:monomer ratio at 350 °C (filled symbols adsorption, empty symbols desorption). (**b**) Pore size distribution of the samples synthesized with 10:1 salt: monomer ratio at 350 °C from NL-DFT using the "N_2 on carbon 77 K (slit-pore, NLDFT equilibrium)" model. (**c**) Cumulative pore volume for the batches of of the samples synthesized with 10:1 salt: monomer ratio at 350 °C from NL-DFT using the "N_2 on carbon 77 K (slit-pore, NLDFT equilibrium)" model. (d) N_2 sorption isotherms at 77 K of the sample pBN-CTF-20-350 (**e**) Pore size distribution and the cumulative pore volume of the sample pBN-CTF-20-350 from NL-DFT using the "N_2 on carbon 77 K (slit-pore, NLDFT equilibrium)" model.

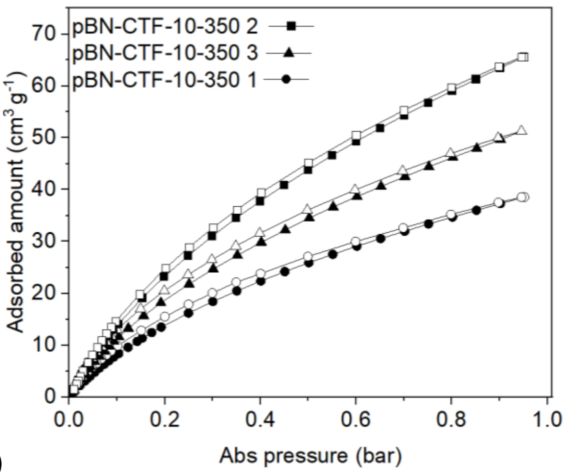

(a)

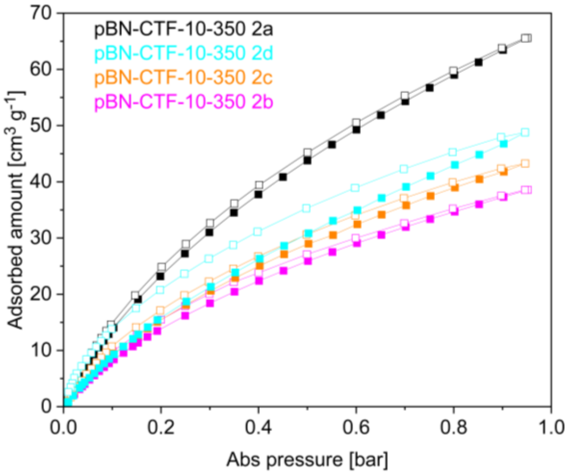

(b)

Figure S6. (a) CO_2 sorption isotherms at 293 K of pBN-CTF-10-350 from three different batches (1, 2, 3). (b) CO_2 sorption isotherms at 293 K of four probes (a, b, c, d) from the same batch 2 of pBN-CTF-10-350 (filled symbols adsorption, empty symbols desorption). See Table S2 for the CO_2 uptake values at 1 bar.

Table S2. Surface area and porosity data from N_2 and CO_2 sorption studies of different pBN-CTF-10-350 samples showing inconsistencies in the sorption behavior.

**CTF-10-350**		**S_{BET} (a) (m^2 g^{-1})**	**V_{tot} (b) (cm^3 g^{-1})**	**V_{micro} (c) (cm^3 g^{-1})**	**V_{micro}/V_{tot} (d)**	**CO_2 uptake 293 K, 1 bar (cm^3 g^{-1}) (e)**	**$V_{1nm}(CO_2)$ (f) (cm^3 g^{-1})**
**Batch 1**		980	0.538	0.377	70	38.5	0.010
**Batch 2**	**probe a**	1027	0.588	0.433	73	65.6	0.013
	**probe b**	-	-	-	-	38.5	0.009
	**probe c**	-	-	-	-	43.3	0.009
	**probe d**	-	-	-	-	48.8	0.008
**Batch 3**		660	0.37	0.30	81	51.3	0.012
**CTF-20-350**		717	0.38	0.29	76	59.6	0.013

(a) Calculated BET surface area from N_2 adsorption at 77 K over a pressure range of P/P_0 = 0.01-0.07. The standard deviation (1σ) for a BET surface area in the range of 1000 m^2 g^{-1} is usually on the order of 20 m^2 g^{-1}.

(b) Total pore volume from N_2 adsorption isotherm at 77 K at P/P_0 =0.95 for pores smaller than 40 nm.

(c) Micropore volume from the NL-DFT method using the N_2 adsorption isotherm at 77 K at P/P_0 = 0.1 for pores with d ≤ 2 nm (20 Å).

(d) Micropore volume/total pore volume.

(e) A standard deviation (1σ) for gas uptake measurements should be at the most 5%.

(f) Pore volume for pores with diameters smaller than 1 nm from CO_2 adsorption isotherms at 293 K and the CO_2 NL-DFT model.

Section S5. CO_2 adsorption isotherms.

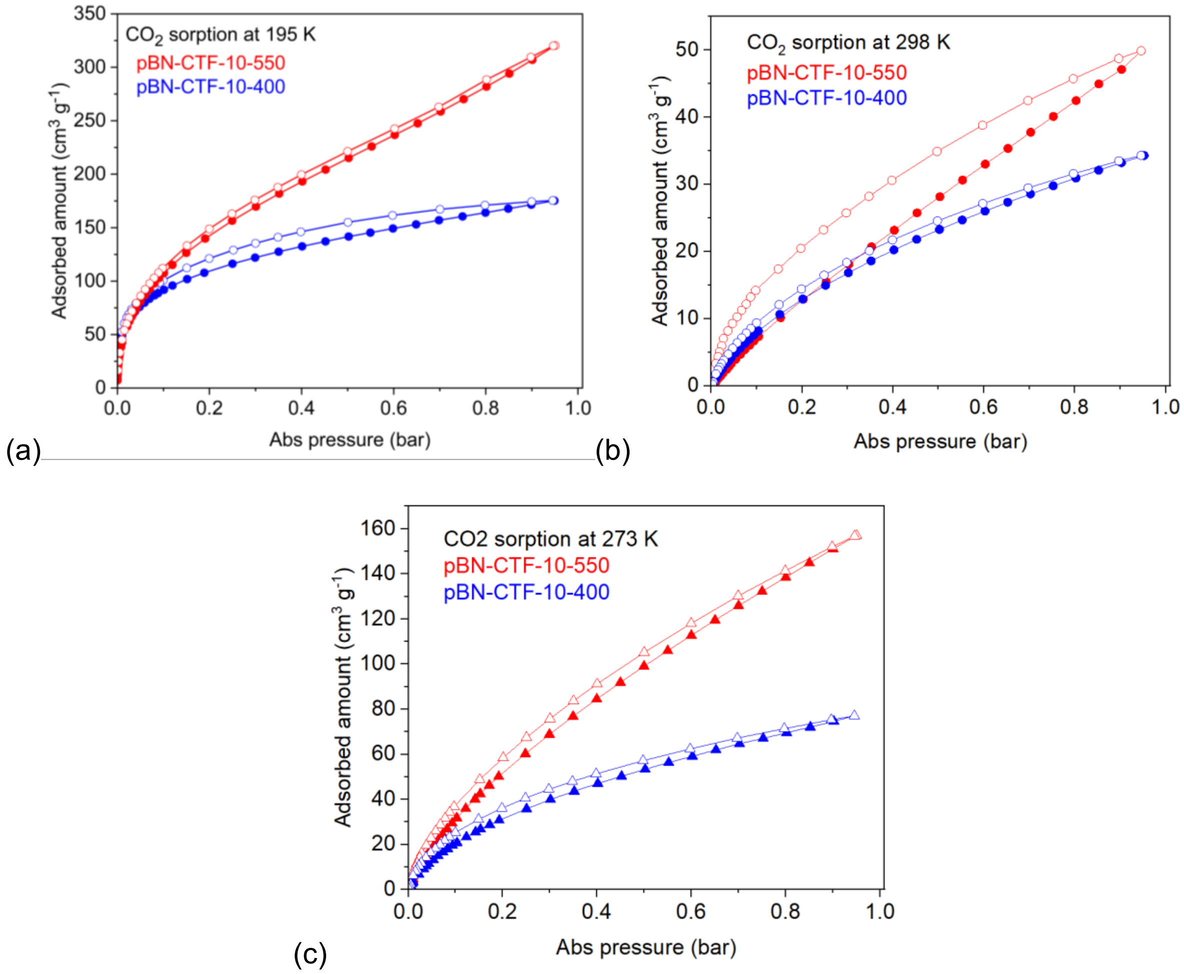

Figure S7. CO_2 adsorption isotherms of pBN-CTF-10s at (a)195 K, (b) 298 K and (c) 273 K.

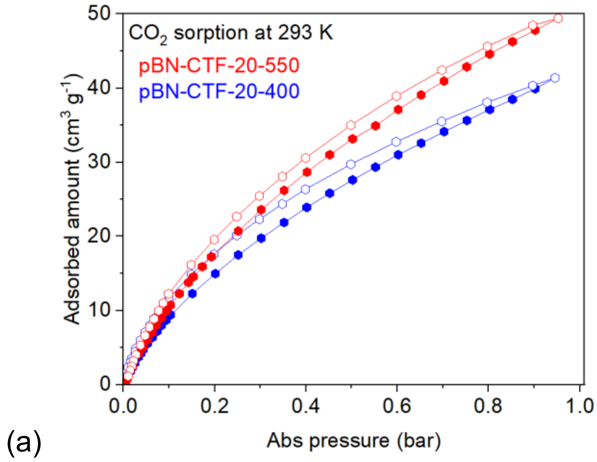

Figure S8. CO_2 adsorption isotherms of pBN-CTF-20-400 and pBN-CTF-20-550 at 293 K.

Table S3. CO_2 uptake results for pBN-CTF-20-400, 550.

Sample	Temp. CO_2 ads. (K)	CO_2 uptake at 1 bar (mmol g^{-1})	CO_2 uptake at 1 bar (cm^3 g^{-1}) [(a)]
**pBN-CTF-20-400**	293	1.7	41.3
**pBN-CTF-20-550**	293	2.0	49.5

[(a)] Conversion between uptake in cm^3 g^{-1} and uptake in mmol g^{-1} at 293 K:
cm^3 g^{-1} = mmol g^{-1} × 24.360 cm^3 $mmol^{-1}$ (i.e. 24.360 L mol^{-1} for an ideal gas at 1 bar and 293 K).

In the main text we had noted that the increase in CO_2 uptake correlates with the increase in BET surface area from N_2 sorption. The CO_2 uptake was measured at the temperatures of 298 K, 293 K, 283 K, 273 K and 195 K (Table 3, Table S4) for the two materials pBN-CTF-10-400 and pBN-CTF-10-550. As we have only two data points for the BET surface area for the all these CO_2 uptake measurements a correlation coefficient for a line through two data points is not meaningful. But we can note that the five lines have all positive slopes and show the increasing CO_2 uptake with BET surface area (Figure S9). Although for the three highest temperatures 283 K, 293 K and 298 K the increase is rather small. A sizable increase is, however, seen at 195 K and 273 K.

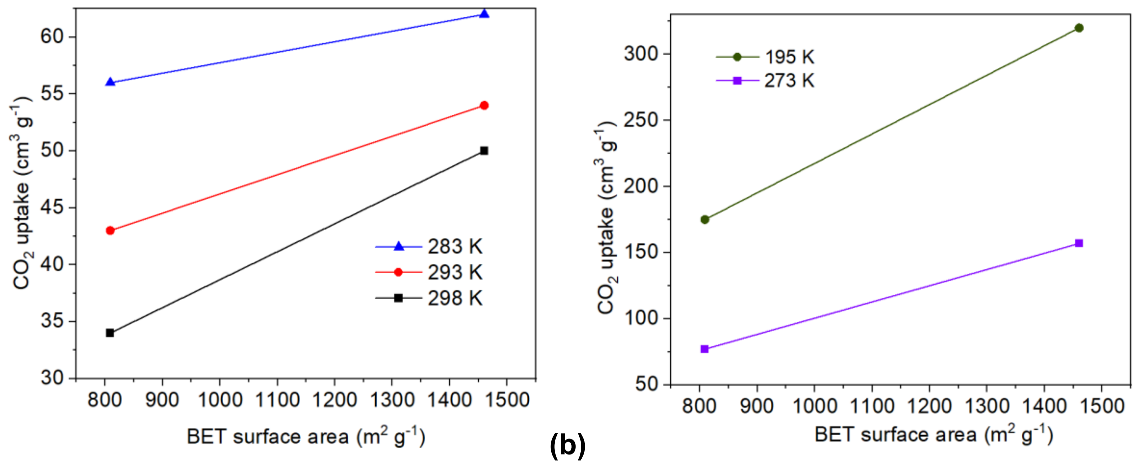

(a) (b)

Figure S9. Scatter plot for the correlation of CO_2 uptake at (**a**) 283 K, 293 K, 298 K, (**b**) 195 K and 273 K and the BET surface area from N_2 sorption for pBN-CTF-10-400 (S_{BET} = 809 m^2 g^{-1}) and pBN-CTF-10-550 (S_{BET} = 1460 m^2 g^{-1}) (Table 2).

If we combine the pBN-CTF-20-400 and -20-550 materials (Table S3) with pBN-CTF-10-400 and pBN-CTF-10-550 for the CO_2 uptake data measured at 293 K (Table S4), then we can correlate the CO_2 uptake over four BET surface areas (Figure S10).

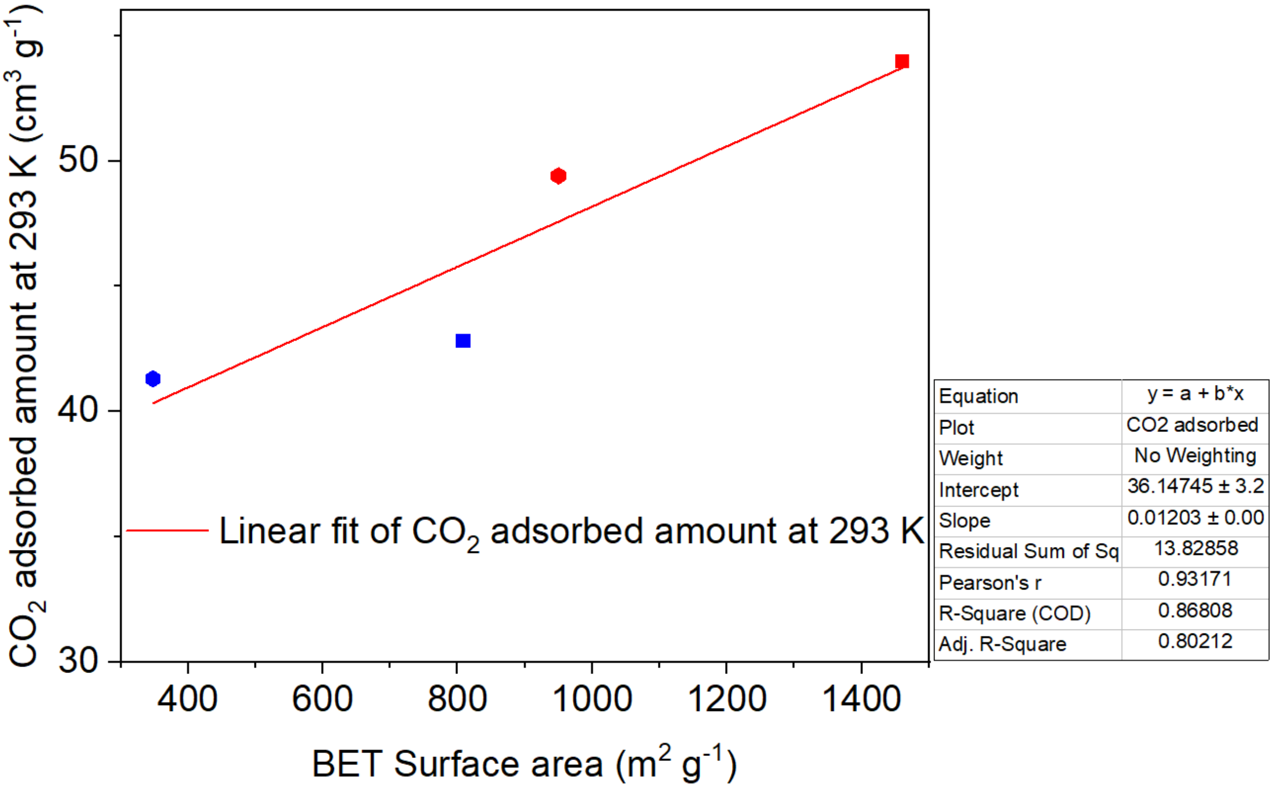

Figure S10. Scatter plot for the correlation of CO_2 uptake at 293 K and the BET surface area from N_2 sorption for pBN-CTF-20-400 (S_{BET} = 348 m^2 g^{-1}), pBN-CTF-10-400 (S_{BET} = 809 m^2 g^{-1}), pBN-CTF-20-550 (S_{BET} = 950 m^2 g^{-1}) and pBN-CTF-10-550 (S_{BET} = 1460 m^2 g^{-1}) (Table 2).

Table S4. Literature CO_2 uptake results.

Sample	Temp. CO_2 ads. (K)	CO_2 uptake at 1 bar (mmol g^{-1})	CO_2 uptake at 1 bar (cm^3 g^{-1}) [(a)]	CTF synthesis temperature (°C)	Ref.
**pBN-CTF-10-400**	273	3.1	77.0	400	This work
**pBN-CTF-10-550**	273	6.4	157.0	550	This work
**pBN-CTF-10-400**	293	1.91	42.8	400	This work
**pBN-CTF-10-550**	293	2.41	54.0	550	This work
**pBN-CTF-10-400**	298	1.37	34.2	400	This work
**pBN-CTF-10-550**	298	2.01	49.9	600	This work
**DCBP-CTF-1**	298	2.07	51.3	400	3
**DCBP-CTF-2**	298	1.84	45.6	400	3
**F-DCBP-CTF-1**	298	3.82	94.7	400	3
**F-DCBP-CTF-2**	298	3.02	74.7	400	3
**F-CTF-1**	298	3.21	79.5	400	4
**F-CTF-1-600**	298	3.41	84.5	600	4
**CTF-DCN-400**	298	1.2	29.7	400	5
**CTF-DCN-500**	298	1.59	39.4	500	5
**CTF-FUM-350**	298	2.38	59.0	350	5
**CTF-FUM-400**	298	1.84	45.6	400	5
**CTF-FUM-500**	298	1.55	38.4	500	5
**CTF-1**	298	1.41	34.9	400	6
**CTF-1-600**	298	2.24	55.5	600	6
**cCTF-400**	298	1.89	46.8	400	6
**cCTF-450**	298	1.41	34.9	450	6
**cCTF-500**	298	1.81	44.8	500	6
**CTF1**	298	3.32	82.3	400/600 (10 h/ 10 h)	7
**CTF2**	298	2.14	53.0	400/600 (10 h/ 10 h)	7
**CTF3**	298	2.03	50.3	400/600 (10 h/ 10 h)	7
**CTF4**	298	3.83	94.9	400/600 (10 h/ 10 h)	7
**CTF5**	298	3.12	77.3	400/600 (10 h/ 10 h)	7
**CTF-ph**	298	3.05	75.6	400/600	8
**CTF-phHT**	298	2.69	66.6	400/800	8
**CTF-py**	298	3.79	93.9	400/600	8
**CTF-pyHT**	298	4.22	104.6	400/800	8
**bipy-CTF500**	298	3.07	76.1	500	9
**bipy-CTF600**	298	2.95	73.1	600	9
**Pym-CTF500**	298	1.77	43.8	500	9
**Pym-CTF600**	298	2.15	53.3	600	9
**fl-CTF300**	298	0.71	17.6	300	10
**fl-CTF350**	298	2.29	56.7	350	10
**fl-CTF400**	298	1.97	48.8	400	10
**fl-CTF500**	298	1.65	40.9	500	10
**fl-CTF600**	298	1.80	44.6	600	10
**HAT-CTF-450/600**	298	4.8	118.9	450/600 (20 h/ 20 h)	11
**CTF-1**	298	1.65	40.9	400	12
**caCTF-1-700**	298	3.55	88.0	700	12
**PHCTF-4**	298	1.57	38.9	250, 300, 350 (10 h), 400 (20 h)	13
**PHCTF-5**	298	1.34	33.2	250, 300, 350 (10 h), 400 (20 h)	13
**CTF-10-400**	298	1.68	41.1	400	14
**CTF-20-400**	298	2.09	51.8	400	14
**CTF-5-500**	298	1.91	47.3	500	14
**CTF-10-500**	298	1.90	44.5	500	14
**bpim-CTF400**	298	2.46	60.9	400	15
**bpim-CTF500**	298	2.77	68.6	500	15
**CTF-CSU41**	298	1.80	44.6	250, 300, 350 (10 h), 400 (20 h)	16

**PHCTF-8(650)**	298	2.54	62.9	650	17
**CTF-BIB-1**	298	2.32	57.5	500	18
**CTF-BIB-2**	298	2.27	56.2	550	18
**CTF-BIB-3**	298	1.98	49.1	600	18
**acac-CTF-5-500**	298	1.97	48.8	500	19
**acac-CTF-10-500**	298	1.91	47.3	500	19
**df-TzCTF600**	298	4.6	114.0	600	20

[(a)] Conversion between uptake in $cm^3\ g^{-1}$ and uptake in $mmol\ g^{-1}$

at 293 K:

$cm^3\ g^{-1} = mmol\ g^{-1} \times 24.360\ cm^3\ mmol^{-1}$ (i.e. 24.360 L mol^{-1} for an ideal gas at 1 bar and 293 K).

at 298 K:

$cm^3\ g^{-1} = mmol\ g^{-1} \times 24.791\ cm^3\ mmol^{-1}$ (i.e. 24.791 L mol^{-1} for an ideal gas at 1 bar and 298 K).

at 273 K:

$cm^3\ g^{-1} = mmol\ g^{-1} \times 24.414\ cm^3\ mmol^{-1}$ (i.e. 24.791 L mol^{-1} for an ideal gas at 1 bar and 298 K).

Section S6. Calculations and fitting for the isosteric heat of adsorption and IAST selectivity of CO_2 and CH_4

The adsorption isotherms were fitted using the 3PSim software, this software is used in order to calculate and fit adsorption data using different fitting models such as Toth, Henry, Freundlich-Langmuir, multi component Sips and many others. After trying different methods of fitting the best one was Freundlich-Langmuir model using the following equation (1):

$$Q = Q_{max} \cdot kC^c/1+kc^c \qquad (1)$$

where
Q: amount adsorbed [mmol g^{-1}]
Q_{max}: maximal loading [mmol g^{-1}]
C: pressure [bar]
k: affinity constant
c: heterogeneity exponent

The isosteric heat of adsorption was calculated after fitting the adsorption isotherms via the Freundlich-Langmuir fit using the following equation [21].

$$n = a \cdot b \cdot p^c/1+b \cdot p^c \qquad (2)$$

Where n: the adsorbed amount (mmol g^{-1}).
a: the maximal loading (mmol g^{-1})
b: the affinity constant
c: the heterogeneity exponent
the pressure at the giving adsorbed amount can be calculated when rearranging the Freundlich-Langmuir equation to the following form.

$$P(n) = c \cdot \sqrt{(n\ /a \cdot b - n \cdot b)} \qquad (3)$$

The parameters a, b and c were taken from the fitting equation.
The isosteric heat of adsorption was then calculated via the Clausius-Clapeyron equation:

$$\Delta H_{(ads)} \cdot n = -R \cdot \ln(p_2/p_1) \cdot (T_1 \cdot T_2)/(T_2-T_1) \qquad (4)$$

Where $\Delta H_{(ads)}$: the isosteric enthalpy of adsorption in kJ mol^{-1}
T_1: absolute Temperature K (283)
T_2: absolute Temperature K (293)
R: the universal gas constant with the value 8.3145 J K^{-1} mol^{-1}.

According to ref [22], when interpolating the loading n, the enthalpy of adsorption is obtained as a function of a loading.

$$\Delta H_{(ads)} \cdot = -R \cdot m' \qquad (5)$$

$$m' = \ln(p_2/p_1) \cdot (T_1 \cdot T_2)/(T_2-T_1) \qquad (6)$$

$$-\Delta H_{(ads)} = Q_{st} \qquad (7)$$

The IAST (ideal adsorbed solution theory) selectivity calculations were done with the "IAST Sips" isotherm model with two components, the parameters of which were taken from the Freundlich-Langmuir fit done with the 3PSim software and used as input. The molar fraction was set to 0.5 for CO_2 and 0.5 for CH_4. The IAST selectivity was calculated after adjusting the settings to a constant pressure and variable molar fraction of both components. Afterwards the CO_2 selectivity values were plotted against the molar fraction of CH_4. IAST selectivity was calculated using the following formula (8):

$$S = (X_1/X_2)/(Y_1/Y_2) \qquad (8)$$

Where
X_1: absorbed fractions of CO_2
X_2: absorbed fractions of CH_4
Y_1: molar fraction of CO_2
Y_2: molar fraction of CO_2

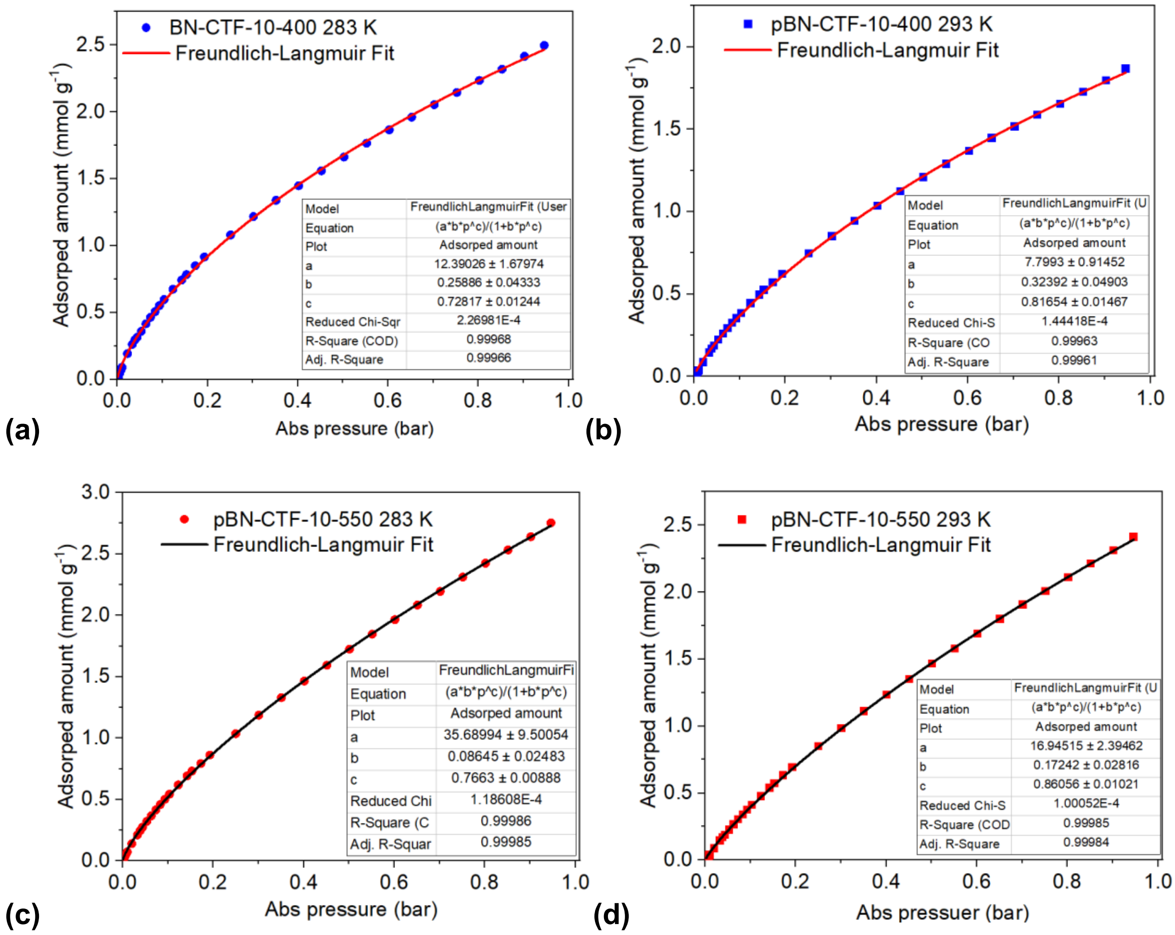

Figure S11. Experimental CO_2 adsorption isotherms of (**a**) pBN-CTF 10-400 at 283 K, (**b**) pBN-CTF 10-400 at 293 K, (**c**) pBN-CTF 10-550 at 283 K and (**d**) pBN-CTF 10-550 at 293 K with their corresponding Freundlich-Langmuir model fits and parameters.

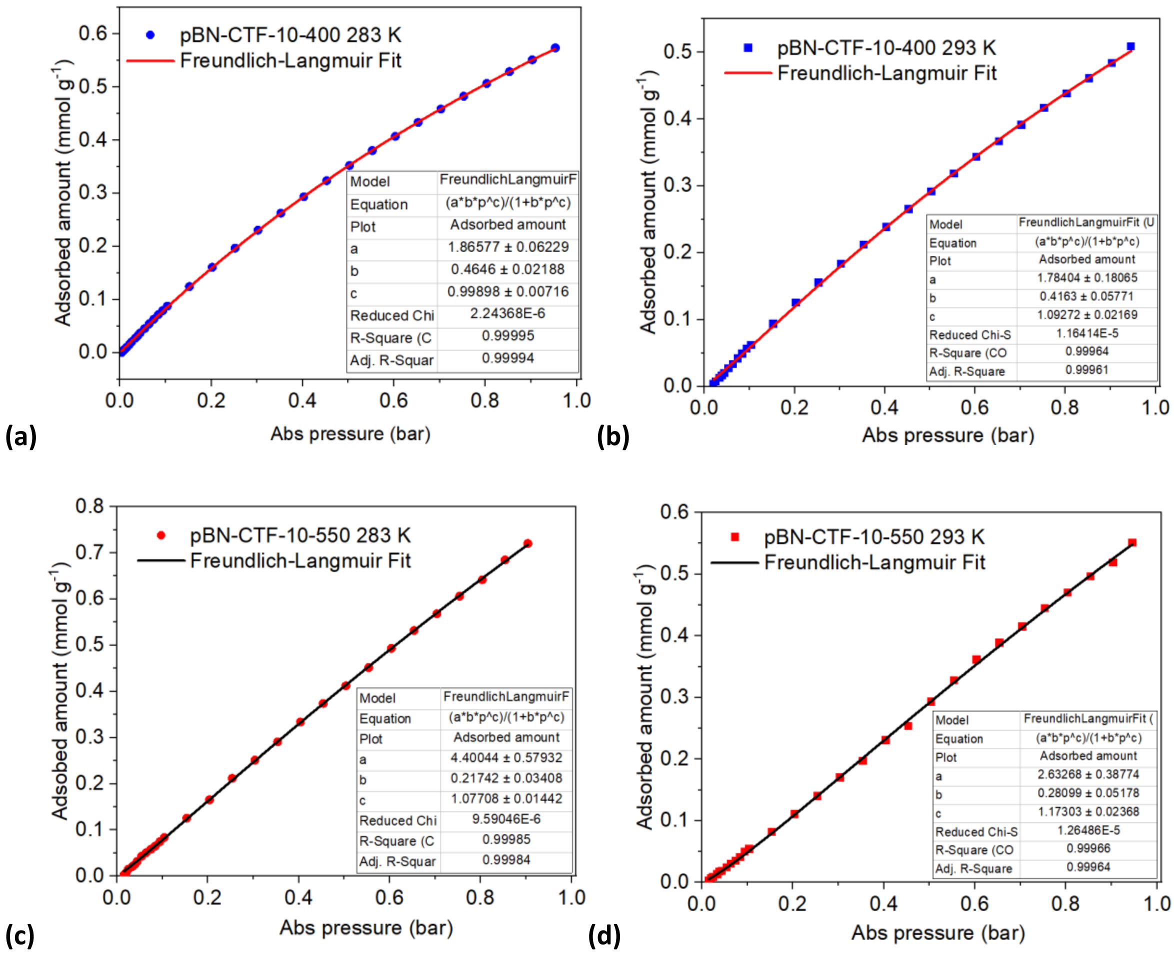

Figure S12. Experimental CH_4 adsorption isotherms of (a) pBN-CTF-10-400 at 283 K, (b) pBN-CTF 10-400 at 293 K, (c) pBN-CTF 10-550 at 283 K, (d) pBN-CTF 10-550 at 293 K, corresponding Freundlich-Langmuir model fits and parameters. The linear CH_4 uptake of pBN-CTF-10-550 did not allow for a meaningful fit.

Table S5. Freundlich-Langmuir adsorption isotherm model fitting parameters for the pBN-CTF-10-400 [(a)]

Sample	R^2	CO_2 Affinity constant (mmol $g^{-1}bar^{-1}$)	Maximal CO_2 loading (mmol g^{-1})	CH_4 Affinity constant (mmol g^{-1} bar^{-1})	Maximal CH_4 loading (mmol g^{-1})
**pBN-CTF-10-400 293 K**	0.999	0.323	7.799	0.416	1.784
**pBN-CTF-10-400 283 K**	0.999	0.258	12.390	0.464	1.8657

[(a)] The linear CH_4 uptake of CTF-10-550 did not allow for a meaningful fit.

Table S6. Comparison between the pBN-CTFs and CTFs from literature for the isosteric heat of adsorption of CO_2 near zero loading (Q_{ads}^0) and the IAST selectivity of CO_2 over CH_4.

Sample	CO_2 Q_{ads}^0 (kJ mol^{-1})	IAST selectivity CO_2/CH_4 (50:50) at 298 K and 1 bar	Ref.
**pBN-CTF-10-400**	79	22	This work
**pBN-CTF-10-550**	60	-	This work
**CTF-DCN-400**	47	8	5
**CTF-DCN-500**	60	10	5
**CTF-Fum-350**	58	20	5
**CTF-Fum-400**	64	20	5
**CTF-Fum-500**	58	14	5
**CTF-1**	27	-	6
**CTF-1-600**	30	-	6
**cCTF-400**	49	-	6
**cCTF-450**	46	-	6
**cCTF-500**	43	-	6
**CTF1**	43	-	7
**CTF2**	23.8	-	7
**CTF3**	25.8	-	7
**CTF4**	21.5	-	7
**CTF5**	24.9	-	7
**CTF-ph**	33.2	-	8
**CTF-phHT**	25.4	-	8
**CTF-py**	35.1	-	8
**CTF-pyHT**	27.1	--	8
**Pym-CTF500**	39	-	9
**Pym-CTF600**	30	-	9
**fl-CTF300**	43	-	10
**fl-CTF350**	32	-	10
**fl-CTF400**	30	-	10
**fl-CTF500**	31	-	10
**fl-CTF600**	32	-	10
**HAT-CTF-450/600**	27	-	11
**CTF-1**	39.6	-	12
**CaCTF-1-700**	30.6	-	12
**PHCTF-4**	24.3	9	13

**PHCTF-5**	23	8	13
**CTF-20-400**	22	-	14
**CTF-5-500**	25	-	14
**bpim-CTF400**	31	-	15
**bpim-CTF500**	28	-	15
**CTF-CSU41**	44	44.6	16
**PHCTF-8(650)**	28	7	17
**CTF-BIB-1**	20.5	6.9	18
**CTF-BIB-2**	20.4	6.9	18
**CTF-BIB-3**	17.4	4.6	18
**acac-CTF-10-500**	23.6	-	19
**df-TzCTF600**	34	30	20

Table S7. BET surface area, CO_2 uptake capacity, Q_{ads}, and CO_2/CH_4 IAST selectivity of the CTFs given in Figure 5 in the main text.

Sample	S_{BET} [$m^2\ g^{-1}$]	CO_2 uptake [$cm^3\ g^{-1}$] [a]	$CO_2\ Q_{ads}^0$ [$kJ\ mol^{-1}$]	IAST CO_2/CH_4	Ref
**pBN-CTF-10-400**	809	34	79	22	This work
**pBN-CTF-10-550**	1460	50	60	- [b]	This work
**HHU-COF-1**	2351	24	- [b]	2	18
**HHU-COF-2**	1346	39	- [b]	2.5	18
**COP-1(N)**	105	22	35	- [b]	53
**COP-S**	253	26	26	- [b]	53
**fl-CTF-400**	2862	49	30	- [b]	59
**fl-CTF-500**	2322	41	31	- [b]	59
**CTF-10-400**	1033	41	28	- [b]	62
**CTF-10-500**	1251	47	26	- [b]	62
**CTF-1**	1034	41	40	- [b]	64

[a] At 298 K. [b] Data not given in reference.

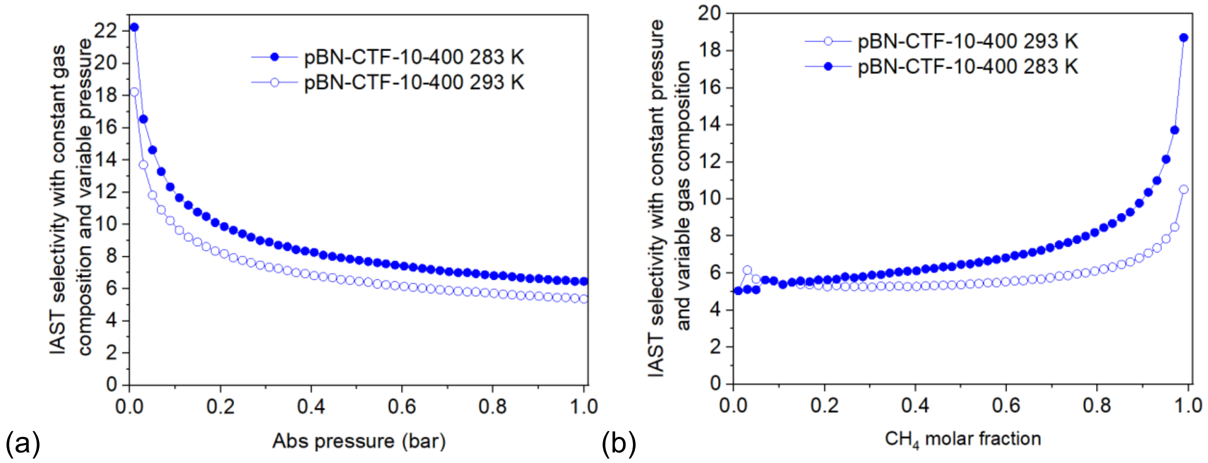

Figure S13. (a) IAST selectivity for pBN-CTF10-400for CO_2 versus CH_4 at a constant gas composition of 0.5:0.5 (mol:mol) and variable pressure at 283 and 293 K. (b) IAST selectivity for pBN-CTF10-400 for CO_2 versus CH_4 at constant gas pressure of 1 bar? and variable gas composition at 283 and 293 K .

Section S7. Thermogravimetric analysis (TGA)

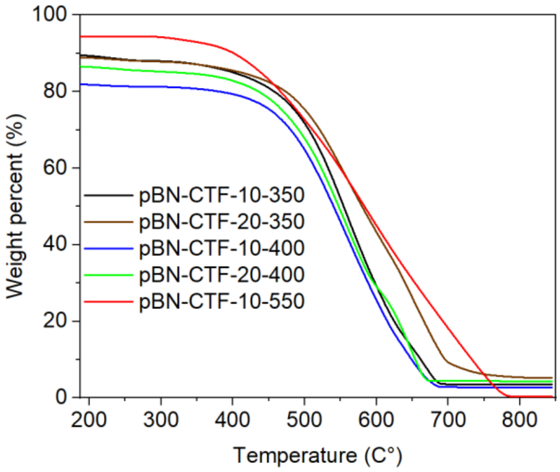

Figure S14. Thermogravimetric analysis of the synthesized pBN-CTFs with a 10 K min^{-1} heating rate under air. An initial loss up to 200 °C is due to adsorbed solvent or moisture.

Section S8. Nuclear magnetic resonance spectrometry (NMR)

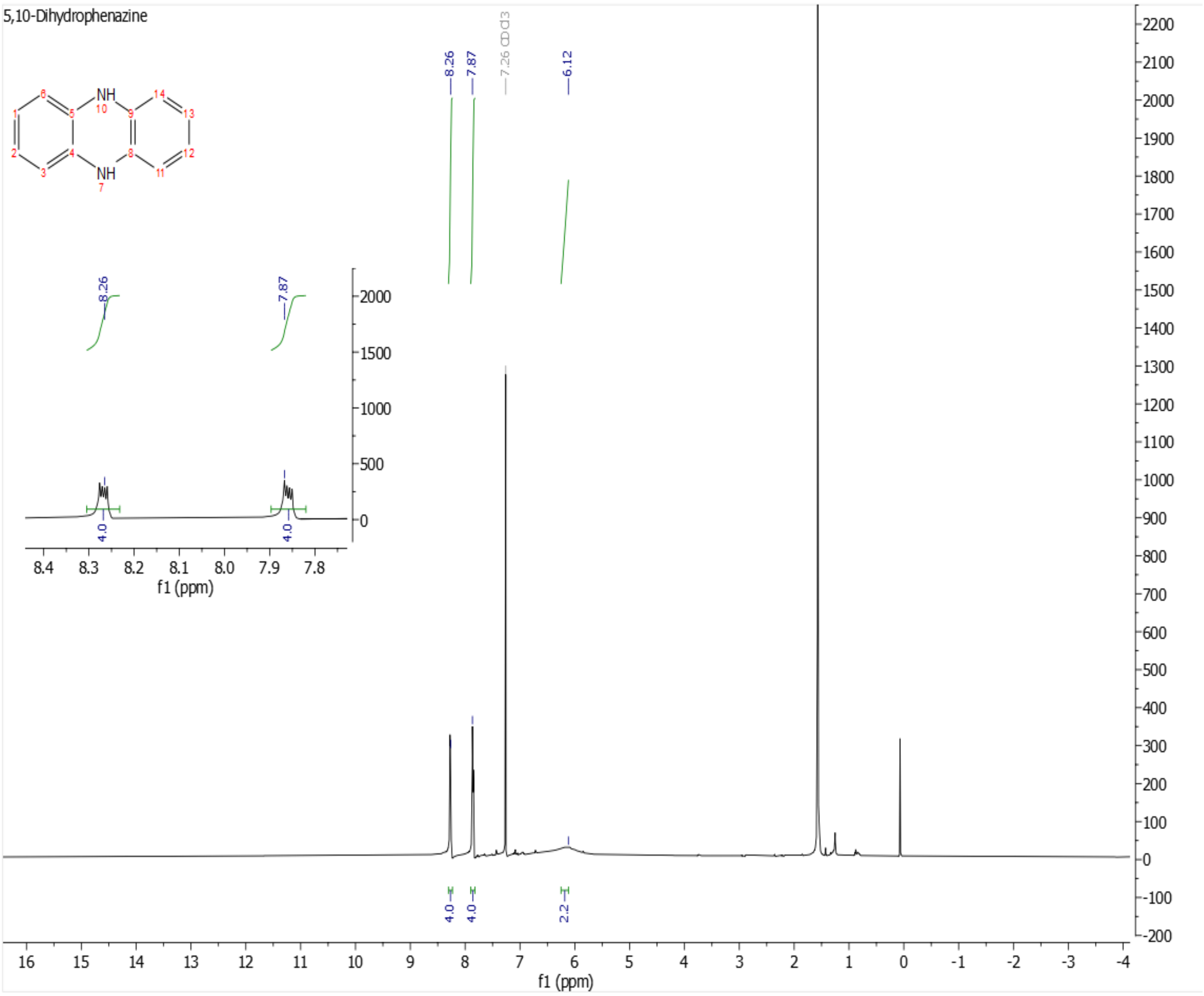

Figure S15. ^{1}H NMR spectrum (600 MHz) of 5,10-dihydrophenazine in $CDCl_3$.

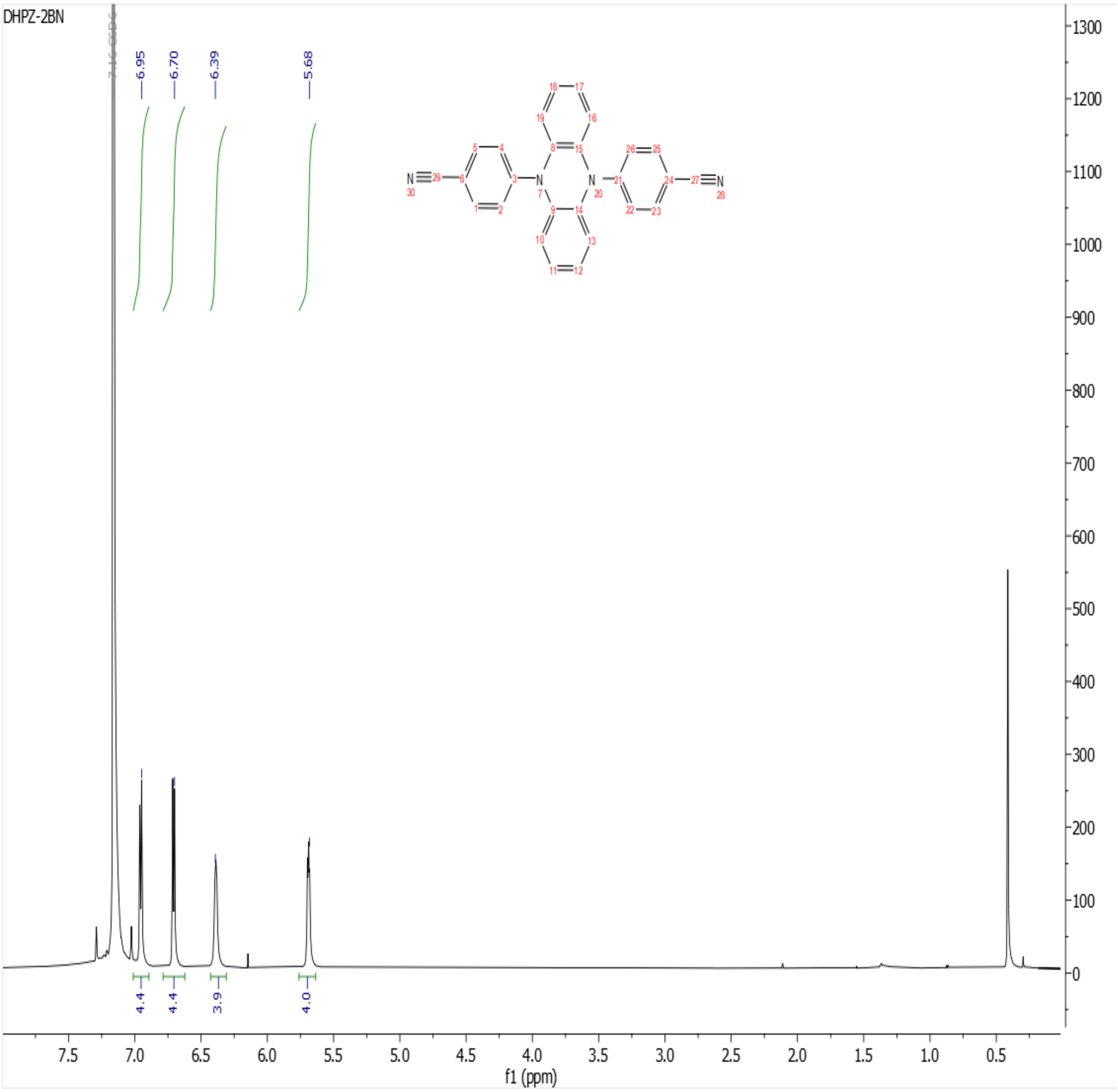

Figure S16. ^{1}H NMR spectrum (600 MHz) of 4,4'-(phenazine-5,10-diyl)dibenzonitrile in C_6D_6.

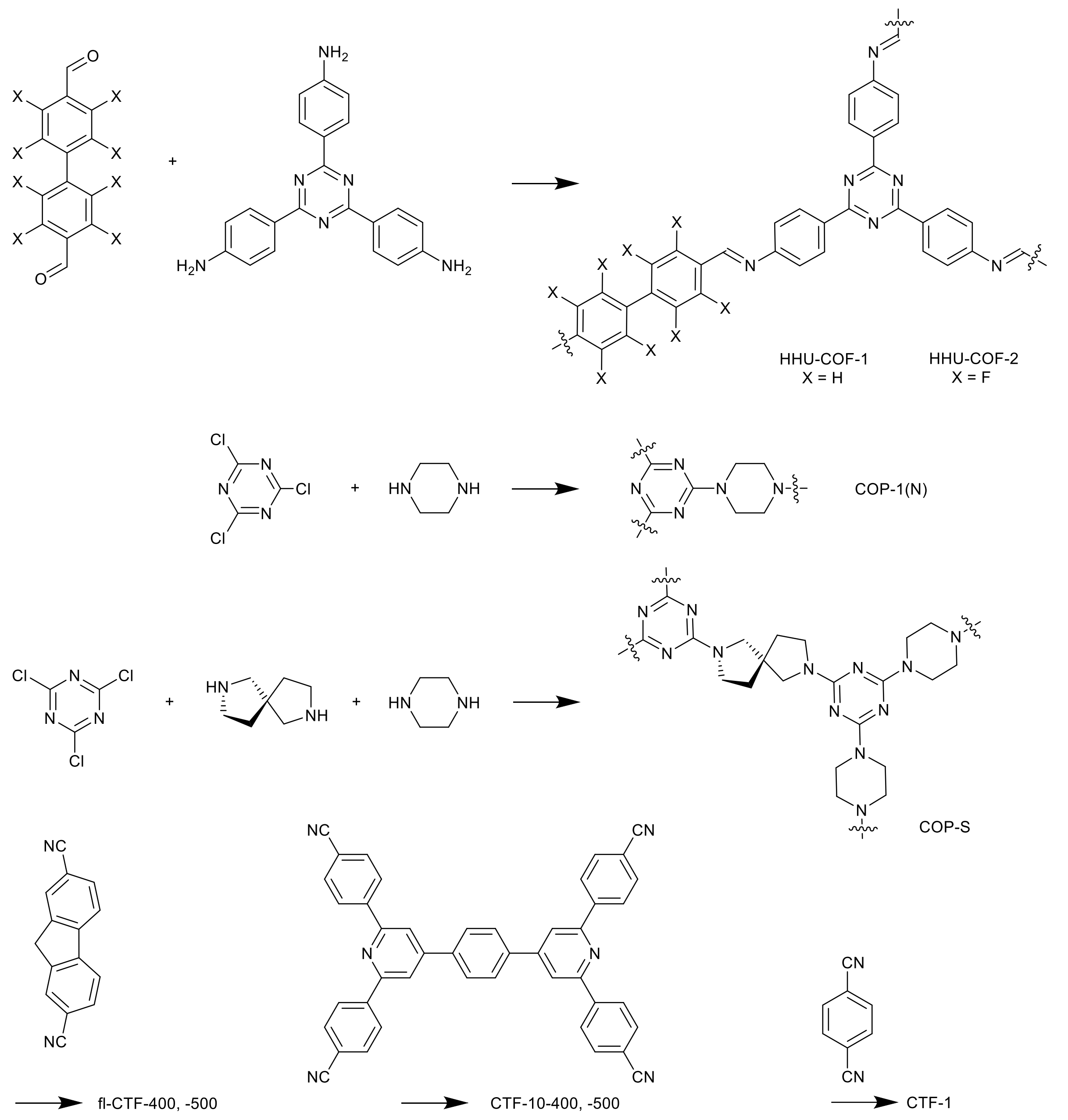

Scheme S1: A graphical presentation of the monomers used for the CTFs in Figure 5.

Section S9. References

1- Bügel, S.; Hähnel, M.; Kunde, T.; de Sousa Amadeu, N.; Sun, Y.; Spieß, A.; Beglau, T.H.Y.; Schmidt, B.M.; Janiak, C. Synthesis and Characterization of a Crystalline Imine-Based Covalent Organic Framework with Triazine Node and Biphenyl Linker and Its Fluorinated Derivate for CO_2/CH_4 Separation. *Materials* **2022**, *15(8)*, 2807.doi: 10.3390/ma15082807

2- Dey, S.; Bhunia, A.; Esquivel, D.; Janiak, C. Covalent triazine-based frameworks (CTFs) from triptycene and fluorene motifs for CO_2 adsorption. *J. Mater. Chem. A* **2016**, *4*, 6259-6263. doi:10.1039/C6TA00638H.

3- Wang, G.; Leus, K.; Jena, H.S.; Krishnaraj, C.; Zhao, S.; Depauw, H.; Tahir, N.; Liu, Y.-Y.; Van Der Voort, P. A fluorine-containing hydrophobic covalent triazine framework with excellent

selective CO_2 capture performance. *J. Mater. Chem. A* **2018**, *6*, 6370-6375, doi:10.1039/C7TA08913A.

4- Zhao, Y.; Yao, K.X.; Teng, B.; Zhang, T.; Han, Y. A perfluorinated covalent triazine-based framework for highly selective and water–tolerant CO_2 capture. *Energy Environ. Sci.* **2013**, *6*, 3684-3692. doi:10.1039/C3EE42548G.

5- Wang, H.; Jiang, D.; Huang, D.; Zeng, G.; Xu, P.; Lai, C.; Chen, M.; Cheng, M.; Zhang, C.; Wang, Z. Covalent triazine frameworks for carbon dioxide capture. *J. Mater. Chem. A* **2019**, *7*, 22848-22870. doi:10.1039/C9TA06847C.

6- Özdemir, J.; Mosleh, I.; Abolhassani, M.; Greenlee, L.F.; Beitle, R.R.; Beyzavi, M.H. Covalent Organic Frameworks for the Capture, Fixation, or Reduction of CO_2. *Front. Energy Res.* **2019**, *7.* doi:10.3389/fenrg.2019.00077.

7- Tuci, G.; Iemhoff, A.; Ba, H.; Luconi, L.; Rossin, A.; Papaefthimiou, V.; Palkovits, R.; Artz, J.; Pham-Huu, C.; Giambastiani, G. Playing with covalent triazine framework tiles for improved CO_2 adsorption properties and catalytic performance. *Beilstein J. Nanotechnol.* **2019**, *10*, 1217-1227. doi:10.3762/bjnano.10.121.

8- Tuci, G.; Pilaski, M.; Ba, H.; Rossin, A.; Luconi, L.; Caporali, S.; Pham-Huu, C.; Palkovits, R.; Giambastiani, G. Unraveling Surface Basicity and Bulk Morphology Relationship on Covalent Triazine Frameworks with Unique Catalytic and Gas Adsorption Properties. *Adv. Funct. Mater.* **2017**, *27*, 1605672. https://doi.org/10.1002/adfm.201605672.

9- Hug, S.; Stegbauer, L.; Oh, H.; Hirscher, M.; Lotsch, B.V. Nitrogen-Rich Covalent Triazine Frameworks as High-Performance Platforms for Selective Carbon Capture and Storage. *Chem. Mater.* **2015**, *27*, 8001-8010. doi:10.1021/acs.chemmater.5b03330.

10- Hug, S.; Mesch, M.B.; Oh, H.; Popp, N.; Hirscher, M.; Senker, J.; Lotsch, B.V. A fluorene based covalent triazine framework with high CO_2 and H_2 capture and storage capacities. *J. Mater. Chem. A* **2014**, *2*, 5928-5936. doi:10.1039/C3TA15417C.

11- Zhu, X.; Tian, C.; Veith, G.M.; Abney, C.W.; Dehaudt, J.; Dai, S. In Situ Doping Strategy for the Preparation of Conjugated Triazine Frameworks Displaying Efficient CO_2 Capture Performance. *J. Am. Chem. Soc.* **2016**, *138*, 11497-11500. doi:10.1021/jacs.6b07644.

12- Lee, Y.J.; Talapaneni, S.N.; Coskun, A. Chemically Activated Covalent Triazine Frameworks with Enhanced Textural Properties for High Capacity Gas Storage. *ACS. Appl. Mater. Interfaces* **2017**, *9*, 30679-30685. doi:10.1021/acsami.7b08930.

13- Yuan, K.; Liu, C.; Zong, L.; Yu, G.; Cheng, S.; Wang, J.; Weng, Z.; Jian, X. Promoting and Tuning Porosity of Flexible Ether-Linked Phthalazinone-Based Covalent Triazine Frameworks Utilizing Substitution Effect for Effective CO_2 Capture. *ACS. Appl. Mater. Interfaces* **2017**, *9*, 13201-13212. doi:10.1021/acsami.7b01783.

14- Wang, G.; Leus, K.; Zhao, S.; Van Der Voort, P. Newly Designed Covalent Triazine Framework Based on Novel N-Heteroaromatic Building Blocks for Efficient CO_2 and H_2 Capture and Storage. *ACS. Appl. Mater. Interfaces* **2018**, *10*, 1244-1249. doi:10.1021/acsami.7b16239.

15- Park, K.; Lee, K.; Kim, H.; Ganesan, V.; Cho, K.; Jeong, S.K.; Yoon, S. Preparation of covalent triazine frameworks with imidazolium cations embedded in basic sites and their application for CO_2 capture. *J. Mater. Chem. A* **2017**, *5*, 8576-8582. doi:10.1039/C6TA11226A.

16- Fu, Y.; Wang, Z.; Li, S.; He, X.; Pan, C.; Yan, J.; Yu, G. Functionalized Covalent Triazine Frameworks for Effective CO_2 and SO_2 Removal. *ACS. Appl. Mater. Interfaces* **2018**, *10*, 36002-36009. doi:10.1021/acsami.8b13417.

17- Yuan, K.; Liu, C.; Liu, C.; Zhang, S.; Yu, G.; Yang, L.; Yang, F.; Jian, X. Construction of triphenylamine functional phthalazinone-based covalent triazine frameworks for effective CO_2 capture. *Polym. J* **2018**, *151*, 65-74. doi: 10.1016/j.polymer.2018.07.061.

18- Du, J.; Liu, Y.; Krishna, R.; Yu, Y.; Cui, Y.; Wang, S.; Liu, Y.; Song, X.; Liang, Z. Enhancing Gas Sorption and Separation Performance via Bisbenzimidazole Functionalization of Highly Porous Covalent Triazine Frameworks. *ACS. Appl. Mater. Interfaces* **2018**, *10*, 26678-26686. doi:10.1021/acsami.8b08625.

19- Jena, H.S.; Krishnaraj, C.; Wang, G.; Leus, K.; Schmidt, J.; Chaoui, N.; Van Der Voort, P. Acetylacetone Covalent Triazine Framework: An Efficient Carbon Capture and Storage Material and a Highly Stable Heterogeneous Catalyst. *Chem. Mater*. **2018,** *30*, 4102-4111. doi:10.1021/acs.chemmater.8b01409.

20- Mukherjee, S.; Das, M.; Manna, A.; Krishna, R.; Das, S. Newly designed 1,2,3-triazole functionalized covalent triazine frameworks with exceptionally high uptake capacity for both CO_2 and H_2. *J. Mater. Chem. A* **2019**, *7*, 1055-1068. doi:10.1039/C8TA08185A.

21- Nuhnen, A.; Janiak, C. A practical guide to calculate the isosteric heat/enthalpy of adsorption via adsorption isotherms in metal–organic frameworks, MOFs. *Dalton Trans.* **2020**, *49*, 10295-1030. doi:10.1039/D0DT01784A.
